# Thromboinflammatory response is increased in pancreas transplant alone versus simultaneous pancreas-kidney transplantation and early pancreas graft thrombosis is associated with complement activation

**DOI:** 10.3389/fimmu.2023.1044444

**Published:** 2023-03-29

**Authors:** Kristina Rydenfelt, Gisle Kjøsen, Rune Horneland, Judith Krey Ludviksen, Trond Geir Jenssen, Pål-Dag Line, Tor Inge Tønnessen, Tom Eirik Mollnes, Håkon Haugaa, Søren Erik Pischke

**Affiliations:** ^1^ Department of Anesthesia and Intensive Care Medicine, Division of Emergencies and Critical Care, Oslo University Hospital, Oslo, Norway; ^2^ Institute of Clinical Medicine, University of Oslo, Oslo, Norway; ^3^ Department of Research & Development, Division of Emergencies and Critical Care, Oslo University Hospital, Oslo, Norway; ^4^ Department of Transplantation Medicine, Section of Transplantation Surgery, Oslo University Hospital, Oslo, Norway; ^5^ Research Laboratory, Nordland Hospital, Bodø, Norway; ^6^ Department of Transplantation Medicine, Section of Nephrology, Oslo University Hospital, Oslo, Norway; ^7^ Department of Immunology, Oslo University Hospital, and University of Oslo, Oslo, Norway; ^8^ Centre of Molecular Inflammation Research, Norwegian University of Science and Technology, Trondheim, Norway; ^9^ Department of Intensive Care Nursing, Lovisenberg University College, Oslo, Norway

**Keywords:** thromboinflammation, pancreas transplant alone, simultaneous pancreas-kidney transplantation, pancreas graft thrombosis, complement, cytokines, coagulation

## Abstract

**Background:**

Pancreas transplant alone (PTA) recipients are more affected by pancreas graft thrombosis, and graft loss compared to simultaneous pancreas-kidney (SPK) recipients. The pathophysiology is unknown, but an increased immune response has been suggested in the PTA recipients. In this observational study, we compared perioperative thromboinflammation between PTA (n=32) and SPK (n=35) recipients, and between PTA recipients with (n=14) versus without (n=18) early graft thrombosis.

**Methods:**

We measured C-reactive protein (CRP), plasma markers of activated coagulation and complement, and cytokines preoperatively and daily during the first postoperative week.

**Results:**

Preoperatively, coagulation and complement activation markers were comparable between PTA and SPK recipients, while cytokine concentrations were higher in SPK recipients (TNF, IL-8, IP-10, MCP-1, MIP-1α; all *p*<0.05). On the first postoperative day, PTA recipients had higher coagulation activation, measured as thrombin-antithrombin complex (TAT), than SPK recipients (*p*=0.008). In the first postoperative week, PTA recipients showed higher relative cytokine release (IL-6, IL-8, G-CSF, IP-10, MCP-1, and MIP-1α; all *p*<0.05) while SPK recipients showed higher absolute cytokine concentrations (TNF, IL-1ra, IL-8, MIP-1α, and IL-4; all *p*<0.05). PTA and SPK recipients showed similar terminal complement complex (TCC, sC5b-9) activation. On the first postoperative day, TCC (OR 1.2 [95% CI 1.0-1.5] for 0.1 CAU/ml increase, *p*=0.02) and CRP (OR 1.2 [95% CI 1.0-1.3] for 10 mg/L increase, *p*=0.04) were associated with an increased risk of early graft thrombosis. TCC was specific for graft thrombosis, while CRP increased with several complications. PTA recipients with compared to those without graft thrombosis had higher TCC pre- (*p*=0.04) and postoperatively (*p*=0.03).

**Conclusion:**

The relative increase in postoperative thromboinflammatory response was more pronounced in PTA recipients. Complement activation was associated with an increased risk of graft thrombosis. This study indicates that innate immune activation rather than elevated levels may affect early postoperative pancreas graft thrombosis.

**Clinical trial registration:**

https://clinicaltrials.gov/ct2/show/NCT01957696, identifier NCT01957696

## Introduction

Pancreas transplantation is currently the only definite treatment for type 1 diabetes mellitus, restoring the body’s glycemic control. The indication is brittle diabetes, and transplantation is performed either as a pancreas transplant alone (PTA) in patients with preserved kidney function or more commonly, as a simultaneous pancreas-kidney (SPK) transplant in patients with a concurrent end-stage renal failure (1).

SPK recipients have approximately 90% 1-year and 80% 5-year graft survival, compared to 75% 1-year and 50% 5-year graft survival for PTA recipients (2–5). The pathophysiology behind this survival gap is poorly understood, but a more active immune system in PTA recipients and a lack of pancreas-specific rejection markers have been suggested (6, 7). Graft thrombosis, the most common cause of early graft loss, is reported to be more frequent in PTA recipients (5, 8, 9). Many risk factors for pancreas graft thrombosis have been identified, but the pathogenesis remains to be explained (9, 10). In the transplantation setting, the ischemia-reperfusion injury triggers a response involving an interplay between several plasma cascades, including complement, coagulation, and fibrinolytic systems, leading to thromboinflammation (11).

C-reactive protein (CRP), one of the most commonly measured markers of inflammation, has been demonstrated to predict several pancreas graft-related complications (12). To explore the thromboinflammatory response further, activation markers of different plasma cascades must be measured. The thrombin-antithrombin (TAT) complex, can be used as a marker of activated coagulation. It is formed to regulate blood coagulation when large amounts of thrombin are generated; thrombin is a potent platelet agonist that cleaves fibrinogen to form a fibrin clot (13). For the complement cascade, C3 is an early marker in the common cascade that can be activated by any of three major activation pathways. C3 in turn activates C5, leading to the formation of the highly pro-inflammatory anaphylatoxin C5a, and the end product, the terminal C5b-9 complement complex (TCC) is shown to be a robust marker of complement activation (14). There are several examples of crosstalk between the coagulation and complement systems (15–17). The complement system, being a part of the innate immune system, plays a major role in the defense against infections, but can also activate coagulation factors and stimulate platelets leading to thrombosis formation (11, 18, 19). The thromboinflammatory response includes the release of cytokines, and the activation of leukocytes, platelets, and endothelial cells (20). After surgery, cytokines are necessary for the defense against infections and for wound healing (21). However, a dysregulation can lead to tissue damage, and an imbalance between pro- and anti-inflammatory cytokines may be involved in the pathophysiology of venous thromboembolism (22).

The nature of the thromboinflammatory response after a solid organ pancreas transplantation, its involvement in pancreas graft thrombosis, and potential differences between PTA and SPK recipients are not yet known. We hypothesized that the perioperative systemic thromboinflammatory response is more pronounced in PTA versus SPK recipients, and between PTA recipients with versus without early graft thrombosis.

## Materials and methods

### Study design, patients, and ethics

We conducted a prospective, observational, single-center study. During a pre-specified 3-year period, from April 2015 to March 2018, all patients (>18 years) admitted for solid-organ pancreas transplantation, as PTA or SPK transplantation, to the Oslo University Hospital in Norway were eligible for study inclusion. Because of very few pancreas after kidney (PAK) transplantations, these were excluded. The study presented is a sub-study of the Norwegian pancreas transplantation study (clinicaltrials.gov NCT01957696, South-Eastern Norwegian ethical committee 2012/2278). Written informed consent was obtained from all participants and we report according to the STROBE checklist (**Figure S1**).

The primary outcome was perioperative differences in thromboinflammatory plasma markers between PTA and SPK recipients. Secondary outcomes were differences in thromboinflammatory plasma markers in PTA recipients with and without graft thrombosis. To describe the postoperative course of our cohort, we registered clinically verified pancreas graft complications (graft thrombi, hematomas/bleedings, and pancreatic leakages) within 30 days, and pancreas graft failure, defined as the need for exogenous insulin or explantation of the transplanted organ, within one-year post-transplantation.

### Patient treatment: Surgery, anticoagulation, and immunosuppression

Pancreas grafts were transplanted in a right retrocolic standing position, with a duodenoduodenostomy and vessel anastomoses to the right common iliac artery and the inferior vena cava.

All study participants received identical treatment, apart from a modification in the clinical protocol for anticoagulation. The first 34 patients (18 SPK, 16 PTA) received intraoperative 30 IU/kg unfractionated heparin intravenously (IV) and 2500 IU low molecular weight heparin (LMWH) subcutaneously (SC) 6 hours postoperatively. This was followed by 2500-5000 IU for SPK and 5000-7500 IU for PTA recipients daily from postoperative day (POD) 1. Acetylsalicylic acid 75 mg per orally (PO) was introduced from POD 7. The remaining 33 patients (17 SPK, 16 PTA) received identical intraoperative heparin, but with the addition of 500 mL of dextran 40, 100 mg/mL intraoperatively and on POD 1. LMWH was administered daily, 5000 IU SC, in both PTA and SPK recipients alike, and acetylsalicylic acid, 75 mg PO, was started from POD 3.

Perioperative immunosuppression consisted of methylprednisolone IV (250 mg, 350 mg if >90kg), mycophenolate mofetil IV (1g twice daily), and anti-thymocyte globulin IV (2mg/kg). Additional doses of anti-thymocyte globulin (1mg/kg) were administered during the first ten postoperative days depending on T-cell count, up to a maximum total dose of 6.5 mg/kg. Maintenance immunosuppression consisted of tacrolimus PO aimed at trough plasma concentrations of 10-12 ng/L during the first 8 postoperative weeks and 6-10 ng/L thereafter, mycophenolate PO (1g twice daily), and a daily dose of prednisolone tapered from 20 mg on POD 1 to 7.5 mg once daily on week 8, then subsequently to 5 mg once daily 6 months postoperatively. Immunosuppressive therapies were the same for PTA and SPK recipients.

### Clinical post-operative evaluations

The study protocol included Doppler ultrasound evaluation of the pancreas graft four hours after reperfusion, with a computed tomography if the ultrasound proved inconclusive. In addition, Doppler ultrasound imaging and contrast-enhanced computed tomography (Iomeron, Bracco, Milan, Italy) were performed on POD 5. Additional imaging was performed on clinical indication. All hematomas/bleedings, pancreatic leakages, and venous thrombi, including both occluding and non-occluding thrombi, are reported in this paper, regardless of their need of intervention. Pancreas graft thromboses were evaluated by a senior radiologist according to the CT-grading system proposed by Hakeem (23) as peripheral thrombosis (grade 1), intermediate non-occlusive thrombosis (grade 2), and central occlusive thrombosis (grade 3). A grade 3 was also assigned in case of ultrasound-based diagnosis of an occlusive thrombus and thus unnecessity of CT-examination.

### Plasma samples

Blood samples were obtained preoperatively and daily for the first seven postoperative days. Blood was collected in 4-mL tubes containing ethylenediaminetetraacetic acid (EDTA). Samples were kept on ice for up to 2 hours before they were centrifuged with 3000 x *g*, for 10 min at 4°C. The isolated plasma was thereafter immediately frozen at -70°C until analysis.

### Analyses of inflammatory markers

*Acute phase protein* CRP was analyzed routinely by the Division of Laboratory Medicine at Oslo University Hospital by immune turbidimetry.

*Coagulation marker* thrombin-antithrombin complex (TAT) was quantified using enzyme-linked immunosorbent assay (ELISA) (Siemens Healthineers, Marburg, Germany). Reference range (97.5^th^ percentile) from healthy adults, as reported by the manufacturer.

*Complement activation products* C3bc and sC5b-9, terminal complement complex (TCC), were analyzed with ELISA, in-house assays, with reference range defined based on healthy blood donors and expressed in complement arbitrary units (CAU)/ml as previously described by Bergseth et al. (24).

*Cytokines* were analyzed using a multiplex cytokine assay (Bio-Plex Human Cytokine 27-Plex Panel; Bio-Rad Laboratories Inc., Hercules, CA, USA) containing interleukins, chemokines, interferons, and growth factors: interleukin (IL)-1β, IL-1 receptor antagonist (IL-1ra), IL-2, IL-4, IL-5, IL-6, IL-7, IL-8, IL-9, IL-10, IL-12 (p70), IL-13, IL-15, IL-17A, eotaxin, basic fibroblast growth factor (bFGF), granulocyte colony-stimulating factor (G-CSF), granulocyte-macrophage colony stimulating-factor (GM-CSF), interferon-gamma (IFN-γ), interferon-inducible protein (IP-10), monocyte chemotactic protein (MCP-1), macrophage inflammatory protein (MIP)-1α, MIP-1β, platelet-derived growth factor-BB (PDGF-BB), regulated upon activation T cell expressed and secreted (RANTES), tumor necrosis factor (TNF), and vascular endothelial growth factor (VEGF). The samples were analyzed on a Multiplex Analyzer (Bio-Rad Laboratories) according to instructions from the manufacturer. For cytokines, we used previously published data from healthy blood donors for reference values (25).

Thirteen of the cytokines, IFN- γ, IL-1β, IL-2, IL-9, IL-12, IL-13, IL-17A, PDGF-BB, RANTES, bFGF, eotaxin, GM-CSF and VEGF, were detectable but low, and within the reference for healthy humans, or demonstrated only minor to no variation during the first postoperative week. These were regarded as negative, and no further analyses were performed.

### Statistics

Patient characteristics between groups were compared with the Mann-Whitney U-test for continuous, and the Chi-squared test for categorical parameters. The outcome between groups was compared with the Fisher’s exact test. All inflammatory biomarkers were non-normally distributed. The Mann-Whitney U-test was used to compare the preoperative inflammatory markers between groups. Fold changes were calculated on each postoperative day as ratios compared to preoperative values. Differences in fold changes at POD 1 compared to preoperative values were evaluated with the Sign’s test separately in PTA and SPK groups and compared between groups with the Mann Whitney U-test. Sums of fold changes from postoperative days 1-7, reflecting relative changes over the first postoperative week, were compared between PTA and SPK recipients with the Mann Whitney U-test.

We used log-10 transformed data in mixed model analyses comparing the time courses of the inflammatory markers between PTA and SPK recipients during the first postoperative week. For the whole study sample, four separate linear mixed models with fixed effects of either time (days), group (PTA/SPK), the interaction of time and group, and all three variables combined were performed with patient identification as a random effect. The effect of *time* described the change in concentration of the inflammatory marker over the first postoperative week for all patients, independent of group effect. The effect of *groups* described differences between the SPK and PTA recipients but did not examine variations between groups over time. The *interaction between group and time*, investigated the variation of the inflammatory marker over time in the two groups giving information if changes over time were different between the two groups. Including all dependent variables (time, group, and interaction thereof) in one model enabled post-estimation of estimated marginal means and thus comparison of inflammatory markers at specific time points between groups and within groups. In PTA recipients only, a subgroup analysis was performed comparing the time courses of the inflammatory markers, and kidney function markers, between recipients with and without graft thrombosis. A significant contribution of the explanatory variable (time/group/interaction thereof) was evaluated by the Wald Chi-Squared test in every model. Due to the change in the anticoagulation regime, the effect of the groups (PTA/SPK) was also assessed separately for the first 34 and the following 33 patients.

Univariate logistic regression was used to determine the odds of developing thrombosis for increased concentrations of any of the inflammatory markers preoperatively or on the first postoperative day. This was done in all recipients together, and separately in PTA recipients. Markers associated with graft thrombosis identified in the logistic regression were further analyzed in mixed model analyses to investigate their abilities to distinguish non-event patients from graft thrombosis, pancreatic leakage, and hematoma/bleeding. Univariate logistic regressions and multivariable logistic regressions including group (PTA/SPK) as an independent variable were also performed to investigate the odds for thrombosis. In addition to inflammatory markers, Body Mass Index (BMI), hemoglobin, and pancreas artery flow were included as these factors may constitute potential confounding factors for the development of graft thrombosis. Probability level *p*<0.05 was considered statistically significant.

We used the following software for the statistical analyses and graphical presentation: R (Core Team (2020). R: A language and environment for statistical computing. R Foundation for Statistical Computing, Vienna, Austria. URL https://www.R-project.org/), StataCorp. 2019. *Stata Statistical Software: Release 16*. College Station, TX: StataCorp LLC and GraphPad Prism version 8.0.0 for Windows, GraphPad Software, San Diego, California USA, https://www.graphpad.com.

## Results

### Study cohort and clinical outcome

**Figure 1:**
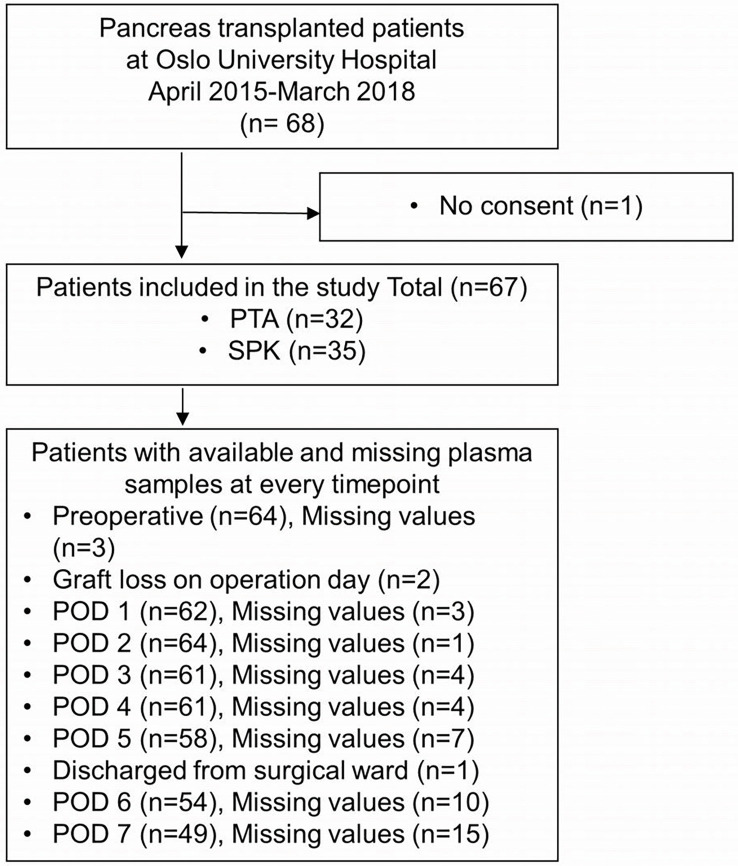
Inclusion of pancreas transplanted patients and available plasma samples throughout the study. PTA, pancreas transplant alone; SPK, simultaneous pancreas-kidney transplantation; POD, postoperative day.

Sixty-seven patients were included, 32 PTA and 35 SPK, providing a total of 473 plasma samples. One patient who did not consent due to language difficulties was excluded (**Figure 1**). Patient characteristics are displayed in **Table 1**. Venous graft thrombosis was diagnosed in 44% of PTA (n=14, 2 peripheral (grade 1), 5 non-occlusive (grade 2), and 7 occlusive (grade 3)), compared to 11% of SPK recipients (n=4, 0 peripheral (grade 1), 1 non-occlusive (grade 2), and 3 occlusive (grade 3) (*p*=0.005, **Table 2**). Graft losses within the first postoperative year were significantly higher in PTA than in SPK recipients (*p*=0.029, **Table 2**). Out of 13 lost grafts, 8 (6 PTA and 2 SPK) had an early graft thrombosis. Arterial pancreas graft thrombi, hematomas/bleedings, and pancreatic leakages did not differ between PTA and SPK recipients (**Table 2**). All arterial pancreas graft thrombi were deemed clinically insignificant and occurred in patients with concurrent venous graft thrombosis. Eight patients (5 SPK, 3 PTA) underwent relaparotomy during the first postoperative week. None of these patients suffered early graft thrombosis.

**Table 1:** PTA and SPK recipient and donor characteristics and perioperative data.

	PTA	SPK	*P*-value
Recipient			
Number of patients	32	35	
Age (years)	40 [34-45]	42 [38-46]	0.47
Male/female	16/16	22/13	0.39
BMI (kg/m^2^)	26.4 [21.5-28.9]	23.9 [22.5-25.7]	0.016
Preoperative blood samples			
Hemoglobin (g/dL)	14.3 [13.4-15.1]	11.4 [10.5-12.3]	<0.001
Thrombocytes (x10^9^/L)	299 [232-362]	256 [239-326]	0.31
INR	1.0 [0.9-1.0]	0.9 [0.9-1.0]	0.020
APTT (s)	32 [31-35]	35 [33-39]	0.0028
Fibrinogen (g/L)^1^	3.3 [2.9-3.8]	4.1 [3.6-4.7]	0.0058
Creatinine (µmol/L)	75 [63-90]	435 [375-639]	<0.001
Urea (mmol/L)	5.9 [4.4-8.4]	11.5 [9.3-18.1]	<0.001
Donor			
Age (years)	25[19-41]	23[20-37]	0.61
Male/female	14/18	20/15	0.34
BMI (kg/m^2^)	22.8 [20.6-24.8]	23.9 [22.5-25.7]	0.12
DBD/DCD (n)	32/0	35/0	
Perioperative data			
Cold ischemia time pancreas (hh:mm)	7:12 [6:11-9:30]	8:53 [7:41-11:02]	0.056
Pancreas artery flow (ml/min)	173 [130-226]	220 [180-290]	0.0074

Continuous variables are presented as median (25th-75th percentile), and categorical variables as numbers. APTT, activated partial thromboplastin time; BMI, body mass index; DBD, donation after brain stem death; DCD, donation after circulatory death; INR, International Normalized Ratio; PTA, Pancreas transplantation alone; SPK, Simultaneous pancreas-kidney transplantation. Fibrinogen was analyzed for 53 patients.

**Table 2:** Clinical outcome in PTA versus SPK.

	PTA (n=32)	SPK (n=35)	*P-*value
Complications within 30 days			
Venous thrombosis	14	4	0.005
Arterial thrombosis	3	5	0.46
Hematoma/bleeding	4	7	0.51
Pancreatic leakage	4	2	0.41
Graft loss			
Graft loss within 30 days	2	3	>0.9
Graft loss within 1 year	10	3	0.029

Fisher’s exact test.

PTA, Pancreas transplantation alone; SPK, Simultaneous pancreas-kidney transplantation.

### Preoperative differences in plasma inflammatory parameters between PTA and SPK recipients

Coagulation marker TAT and complement activation products C3bc and TCC did not differ between PTA and SPK recipients preoperatively. Six of the investigated cytokines: TNF, IL-8, IP-10, MCP-1, MIP-1α and IL-4, were significantly higher in SPK compared to PTA recipients (**Table 3**).

**Table 3:** Preoperative inflammatory parameters in PTA and SPK patients.

Parameter	PTA (n=32)	SPK (n=35)	*P-*value
Acute phase protein			
CRP (mg/L)	0.9[0.6-5.3]	2.4[1.1-4.3]	0.022
Coagulation (µg/L)			
TAT	9.0[5.8-21]	8.8[3.4-20]	0.52
Complement (CAU/ml)			
C3bc	3.7 [2.9-6.2]	3.9[3.0-6.5]	0.90
TCC	0.3[0.2-0.4]	0.3[0.2-0.3]	0.42
Cytokines (pg/ml)			
TNF	22[13-32]	41[29-50]	0.002
IL-6	1.0[0.5-3.4]	2.0[0.9-6.0]	0.11
IL-8	2.2 [1.2-4.7]	4.3 [2.7-7.4]	0.006
IL-1ra	101[71-249]	146[103-287]	0.11
IL-10	2.3[0.8-4.9]	2.9[0.8-11]	0.87
IL-4	2.2[1.1-2.5]	3.0[2.3-3.7]	0.0035
G-CSF	5[1-127]	14[1-158]	0.21
IP-10	373[239-521]	633[445-978]	0.0015
MCP-1	13[6-20]	17[13-23]	0.033
MIP-1α	1.4[0.6-3.9]	2.8[1.6-5.1]	0.049
MIP-1β	97[59-107]	90[76-104]	0.77
IL-5	4.0[3.2-15]	7.3[3.2-21]	0.39
IL-7	2.6[1.1-5.5]	4.8[1.4-14]	0.08
IL-15	16[7.6-24]	21[15-45]	0.18

Values presented as median (25th-75th percentile). Mann-Whitney U-test. Significant differences are highlighted in bold.

CAU, complement arbitrary unit; G-CSF, granulocyte colony-stimulating factor; IL, interleukin; IL-1ra: interleukin-1 receptor antagonist; IP-10, interferon gamma-induced protein 10; MCP-1, monocyte chemoattractant protein 1; MIP, macrophage inflammatory protein; PTA, Pancreas transplantation alone; SPK, Simultaneous pancreas-kidney transplantation; TAT, thrombin-antithrombin complex; TCC, terminal complement complex; TNF, tumor necrosis factor.

### Time course of plasma inflammatory parameters over the first postoperative week for PTA and SPK recipients

**Figure 2:**
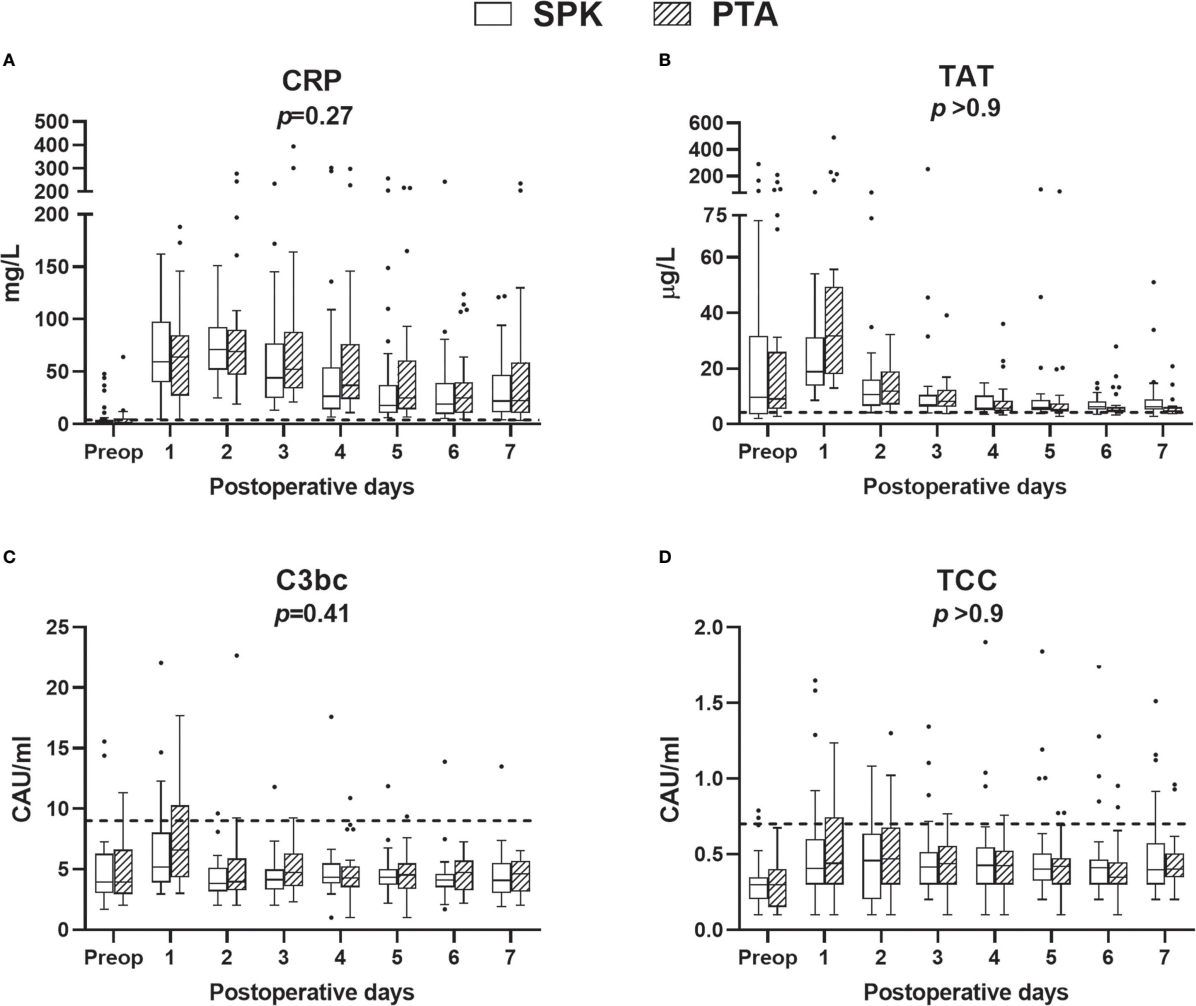
Plasma concentrations of C-reactive protein (CRP) **(A)**, coagulation **(B)**, and complement markers **(C, D)**, preoperatively (preop) and during the first postoperative week, for simultaneous pancreas-kidney transplantation (SPK) (n=35) and pancreas transplant alone (PTA) (n=32) patients. Data are presented as boxplots (line, median; box, interquartile range) with whiskers (25^th^ and 75^th^ percentiles -/+ 1.5 x interquartile range). Dashed lines represent upper reference limits defined previously in samples from healthy blood donors and are given for reference only. Two outliers are not shown, C3bc value 34 and TCC value 3.8, in PTA patients on postoperative day 1. *P*-values represent the-postoperative overall group effect (Wald Chi-Squared test), excluding the preoperative samples, in a linear mixed model. CAU, complement arbitrary unit; CRP, C-reactive protein; TAT, thrombin-antithrombin complex; TCC, terminal complement complex.

**Figure 3:**
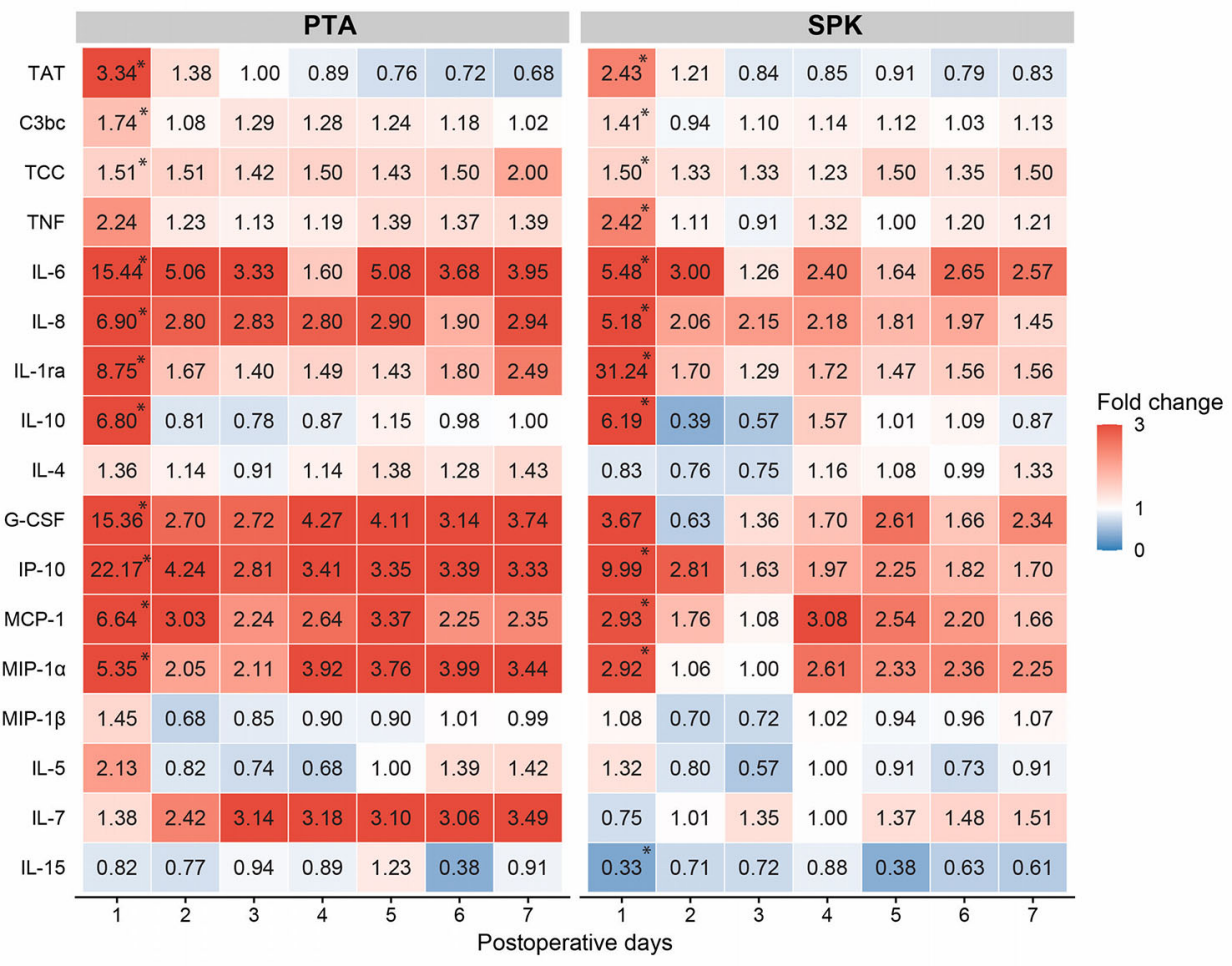
Median fold changes of inflammatory markers for postoperative days 1-7 compared to preoperative values (day=0) for pancreas transplant alone (PTA) and simultaneous pancreas-kidney (SPK) recipients. The red color indicates increased fold change and the blue color decreased fold change compared to preoperative median concentration. *Indicates significantly (*p*<0.05) increased fold change from preoperative values to the first postoperative day (Sign test) within PTA and SPK groups. Fold changes on POD 1 and sums of fold changes POD1-7 were compared between SPK and PTA recipients (Mann Whitney U-test) and presented in Table S4. CRP, C-reactive protein; G-CSF, granulocyte colony-stimulating factor; IL, interleukin; IL-1ra; interleukin-1 receptor antagonist; IP-10, interferon gamma-induced protein 10; MCP-1, monocyte chemoattractant protein 1; MIP, macrophage inflammatory protein; TAT, thrombin-antithrombin complex; TCC, terminal complement complex; TNF, tumor necrosis factor.

CRP, coagulation marker TAT and complement markers C3bc and TCC increased significantly (*p*<0.001 for all) from preoperative values to POD 1 (**Figures 2A–D**, **Table S1**). During the first postoperative week, CRP, TAT, and C3bc decreased, demonstrating considerable time and group-by-time effects (*p*<0.001 for all). TCC remained elevated compared to preoperative values in both groups (**Figure 2D**). The changes over time of these markers did not differ between PTA and SPK recipients (**Tables S1, S2**). TAT, however, was significantly (*p*=0.008) higher on POD 1 (**Table S3**), and summed fold changes for TCC from POD 1 to POD 7 (*p=*0.031) in PTA compared to SPK recipients (**Figure 3**, **Table S4**).

**Figure 4:**
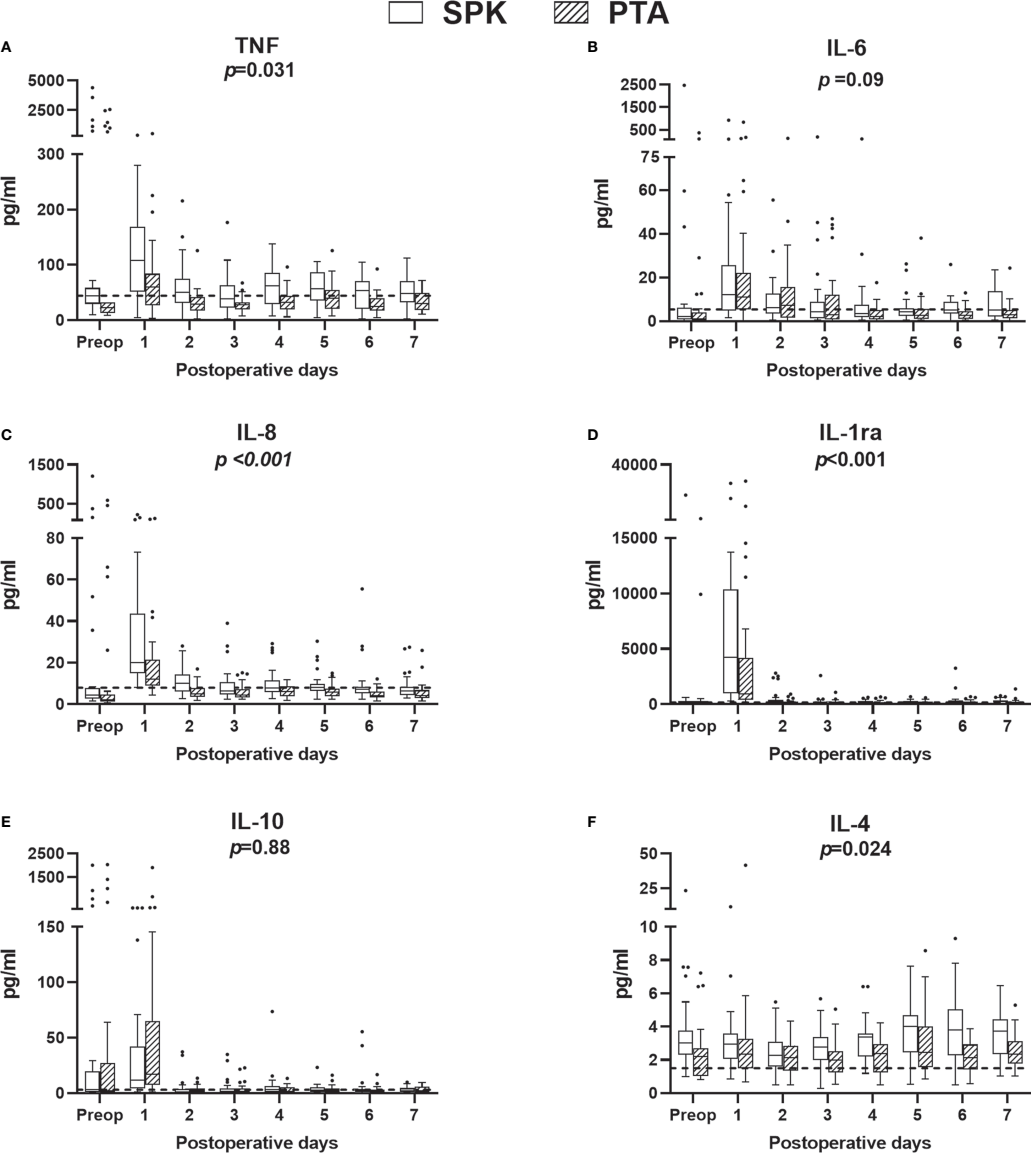
Plasma concentrations of proinflammatory **(A–C)** and anti-inflammatory **(D–F)** cytokine preoperatively (preop) and during the first postoperative week, for simultaneous pancreas-kidney (SPK) (n=35) transplantation and pancreas transplant alone (PTA) (n=32) patients. Boxplots, reference lines, and overall effects of transplantation group are calculated and presented as in **Figure 2**. IL, interleukin; IL-1ra; interleukin-1 receptor antagonist; pg, picogram; TNF, tumor necrosis factor.

**Figure 5:**
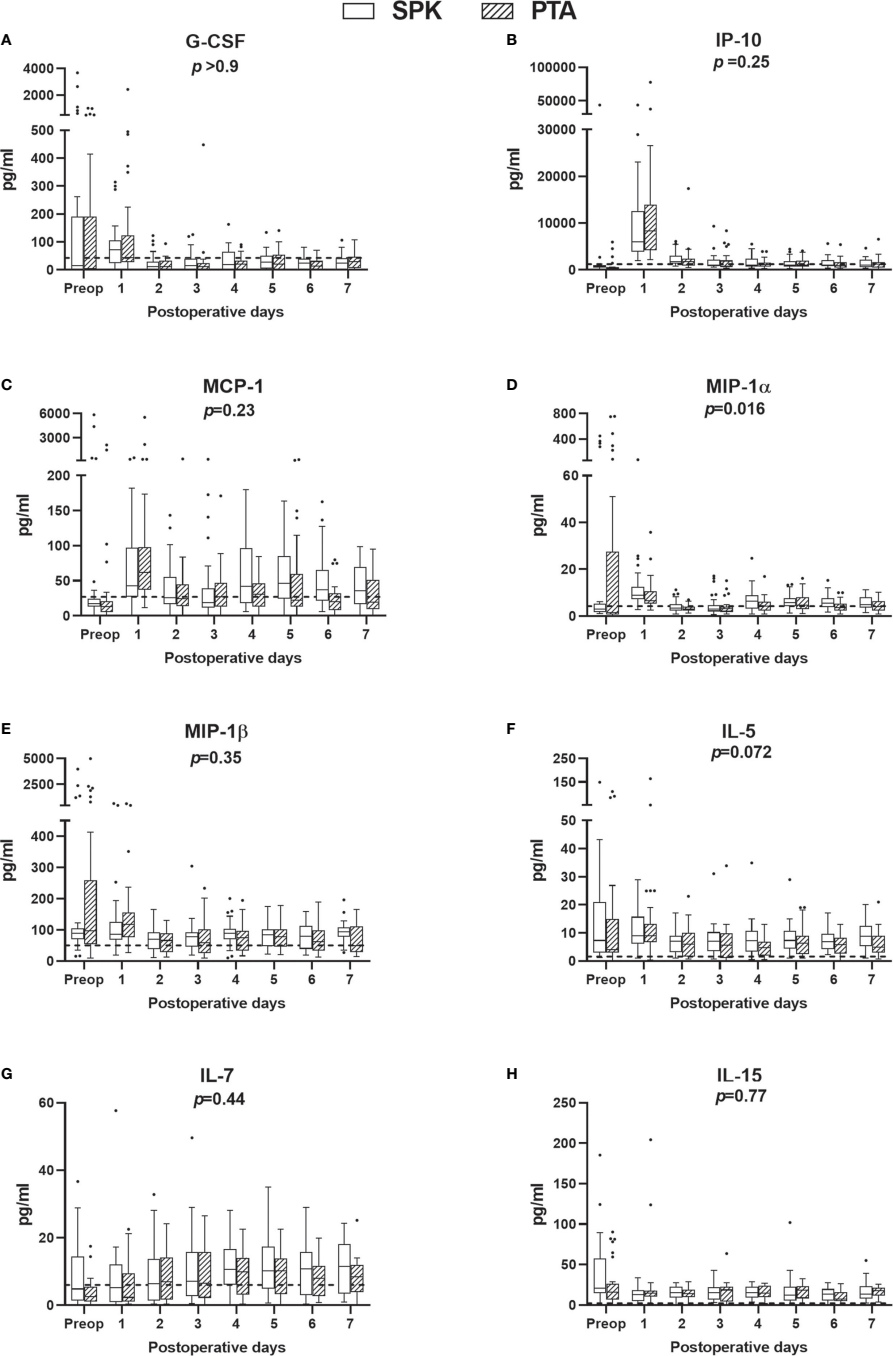
**(A)** Growth factor, chemokines **(B–E)**, and pleiotropic **(F–H)** cytokine plasma concentrations preoperatively (preop) and during the first postoperative week, for simultaneous pancreas-kidney (SPK) (n=35) transplantation and pancreas transplant alone (PTA) (n=32). Boxplots, reference lines, and overall effects of transplantation group were calculated and presented as in **Figure 2**. G-CSF, granulocyte colony-stimulating factor; IL, interleukin; pg, picogram.; IP-10, interferon gamma-induced protein 10; MCP-1, monocyte chemoattractant protein 1; MIP, macrophage inflammatory protein.

Cytokines were grouped according to their main function and subgroups (**Figures 4**, **5**). From preoperative values to POD 1, eight of the fourteen investigated cytokines increased significantly in both PTA and SPK recipients: IL-6, IL-8, IL-1ra, IL-10, G-CSF, IP-10, MCP-1, and MIP-1α (*p ≤* 0.031 for all, **Table S1**). TNF increased significantly (*p*=0.02) only in SPK recipients (**Table S1**). All cytokines demonstrating an initial increase decreased significantly from POD 1 to POD 7 (*p ≤* 0.036 for all, **Figures 4**, **5**, **Table S1**). IL-7 increased significantly over the first postoperative week (*p ≤* 0.017), while there were no discernible time patterns for MIP-1α, IL-4, IL-5, and IL-15 (**Figures 4**, **5**, **Table S1**). Except for IL-15, all cytokines demonstrated postoperative time and time-by-group effects (*p*<0.001 for all, **Table S2**). Five cytokines were elevated in SPK compared to PTA recipients: TNF, IL-8, IL-1ra, MIP1α, and IL-4 (*p ≤* 0.031, **Table S2**). The trend with higher inflammatory markers in SPK recipients was seen both before and after the change in the anticoagulation regime (**Table S4**). Three cytokines had higher concentrations on POD 1 in SPK versus PTA recipients: TNF, IL-8, and IL-1ra (*p ≤* 0.034 for all, **Table S3**). Fold changes from preoperative values to POD 1 were significantly higher in PTA than in SPK recipients for IL-6, IL-4, IP-10, and MCP-1 (*p ≤* 0.03 for all, **Figure 3**, **Table S5**). The changes over the first postoperative week, reflected by summed fold changes from POD 1 to POD 7, were significantly higher in PTA than in SPK recipients for eight of the cytokines: IL-6, IL-8, IL-4, G-CSF, IP-10, MCP-1, MIP-1α and IL-7 (*p*<0.05 for all, **Figure 3**, **Table S5**).

### Risk for thrombotic complications

PTA was associated with a higher risk for venous graft thrombosis formation within 30 days postoperatively, compared to SPK transplantation (OR 6.0 [95% CI 1.7-21.2], *p*<0.001). Of the investigated inflammatory markers, only CRP (OR 1.16 [95% CI 1.01-1.34] for 10 mg/L increase, *p*=0.042) and TCC (OR 1.23 [95% CI 1.04-1.47] for 0.1 CAU/ml increase, *p*=0.019) at the first postoperative day were associated with an increased risk of graft thrombosis (**Table S6**). CRP was significantly increased in patients with early graft thrombosis (*p*=0.008), pancreatic leakage (*p*=0.015), and bleeding/hematoma (*p*=0.007) compared to patients with no events. TCC was significantly increased in patients with graft thrombosis (*p*=0.012), but not in patients with pancreatic leakage (*p*=0.32) or bleeding/hematoma (*p*=0.09).

In univariate logistic regression analyses the potential confounding factors, BMI, hemoglobin, and pancreas artery flow, were all associated with graft thrombosis (**Table S7**). However, BMI, but neither hemoglobin nor pancreas artery flow was associated with an increased risk of graft thrombosis when adjusted for the type of transplantation (SPK/PTA) (**Table S7**). The association between increased TCC on POD 1 with increased risk for graft thrombosis remained when adjusted for the type of transplantation (OR 1.3[1.1-1.5], *p*=0.029) and BMI (OR 1.5[1.0-2.2], *p*=0.009, **Table S8**).

### Subgroup analysis of PTA recipients for thrombotic complication

**Figure 6:**
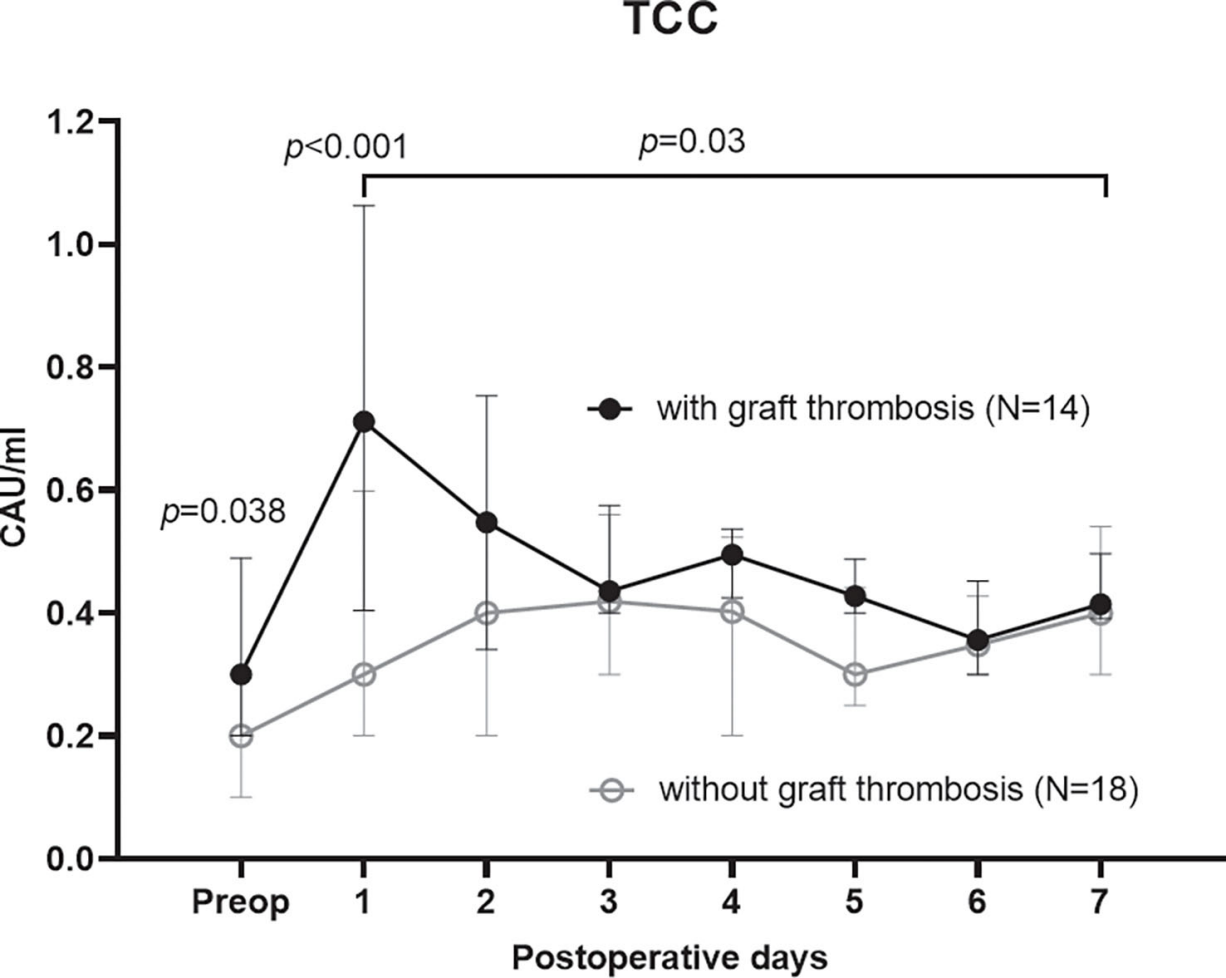
Terminal complement complex (TCC), as a marker of complement activation, in the subgroup analysis of PTA recipients with and without an early postoperative graft thrombosis. TCC was significantly higher in PTA patients with a graft thrombosis both preoperatively (Mann Whitney U-test), on the first postoperative day (mixed model analysis), and as a group effect over the first postoperative week (mixed model analysis).

As most thrombi were detected in PTA recipients, we performed a subgroup analysis to investigate differences between recipients with (n=14) and without graft thrombosis (n=18). The complement activation product TCC was significantly higher preoperatively (*p*=0.038), on POD 1 (*p*<0.001), and demonstrated a significant group effect (*p*=0.03), with overall higher concentrations in thrombotic recipients (**Figure 6**, **Tables S9-S11**). CRP was significantly higher in thrombotic recipients on POD 1 (*p*=0.031). TCC (OR 1.4 [95% CI 1.0-2.0], for 0.1 CAU/ml increase, *p*=0.029) and CRP (OR 1.4 [95% CI 1.1-1.8] for 10 mg/ml increase, *p*=0.021) on POD 1 were associated with an increased risk of thrombosis formation in PTA recipients (**Table S12**). Furthermore, IL-6 (*p*=0.011) was significantly elevated on POD 1, and MIP-1α (*p*=0.016) and IL-5 (*p*=0.035) demonstrated significant group effects in thrombotic recipients (**Table S10, S11**). Pre- and postoperative kidney function measured as creatinine and urea, and perioperative pancreas artery flow did not differ between PTA recipients with and without pancreas graft thrombosis (all *p*>0.1).

## Discussion

To our knowledge, this prospective observational study is the first to compare thromboinflammation, including complement activation, between PTA and SPK recipients. PTA recipients demonstrated an increased coagulation activation early postoperatively, and a relatively higher cytokine release, while the absolute cytokine concentrations were higher in SPK recipients both pre- and postoperatively. Notably, CRP and complement activation as detected by TCC early postoperatively, were associated with an increased risk of pancreas graft thrombosis.

Pancreas graft thrombosis is associated with early graft loss and our total incidence of 27% diagnosed graft thrombosis within the first postoperative month is comparable with previous reports including both partial and complete thrombi (23, 26, 27). Our study cohort had 48% of pancreas transplantations performed as PTA, while most centers report less than 10% (7, 28). The number of graft thrombi leading to early graft loss and the significantly lower 1-year graft survival in PTA than in SPK recipients observed in this study were comparable to internationally published data (5, 7–9, 29).

Preoperatively, TAT and cytokine concentrations were overall higher than reference values, which may be explained by the hypercoagulable state and hyperglycemia-related low-grade chronic inflammation related to diabetes (30, 31). With similar preoperative coagulation and complement activation markers in PTA and SPK recipients, a pre-existing increased thrombogenicity in PTA compared to SPK recipients appears unlikely, even if the platelet dysfunction associated with uremia could be protective for SPK recipients (32). The higher absolute cytokine concentrations found in SPK compared to PTA recipients can be caused by chronic inflammation precipitated by chronic kidney disease and hemodialysis, together with a decreased renal clearance (33, 34).

After transplantation, CRP, the coagulation and complement activation markers, and most cytokines increased in all recipients, most probably due to the ischemia-reperfusion injury (35) and the surgical trauma (36). The PTA recipients demonstrated a more prominent coagulation activation with higher TAT levels on the first postoperative day and higher relative increases of several cytokines, which could correspond to previous suggestions of a “healthier” immune system in the PTA versus the SPK recipients (6, 7). Transplantation of two organs in SPK recipients did not lead to a larger relative increase of cytokines compared to PTA patients, which might be due to uremia-mediated immune dysfunction (37). These differences in the thromboinflammatory response between SPK and PTA recipients warrants further investigations into the potential role of thromboinflammation for the outcome.

Increased postoperative CRP and TCC were the only markers investigated in our study that were associated with an increased risk of early pancreas graft thrombosis. We did not discover any associations between graft thrombosis and increased concentrations of postoperative TAT nor any of the cytokines. Thus, it remains to be proven that short-term TAT increases result in an increased risk of thrombosis in non-cancer patients (38). Cytokines have been reported to regulate local formation and resolution of thrombosis (22) and closer monitoring of cytokines near the graft might thus be necessary to prove this association in pancreas transplantation. CRP has previously been associated with thrombosis formation and an increased risk of thromboembolism in other settings (39–41), but is a non-specific marker that increases with several complications. Our results correspondingly demonstrated increased CRP with graft thrombosis, pancreatic leakage, and bleeding during the first postoperative week, while TCC only increased with graft thrombosis, indicating that complement activation is more specific for graft thrombosis compared to CRP early postoperatively. Complement activation has previously been associated with graft thrombosis in liver transplantation (42), and venous thromboembolism in the general population (43). The association between complement activation and graft thrombosis supports the concept of complement-coagulation cascade cross-talk, but whether the increased complement activation contributes to thrombosis formation, is caused by thrombosis formation or both cannot be answered by our observational study design (11, 17, 19). However, the notion that PTA recipients with graft thrombosis had already pretransplant increased TCC concentrations and that early postoperative increase in TCC was higher in patients with graft thrombosis at any time-point indicate that TCC might be considered as a risk factor for graft thrombosis.

Through the subgroup analysis of PTA recipients, we eliminated potential confounders like uremia-induced inflammation and anticoagulatory effects, which occur more frequently in SPK recipients. Here, we found significantly higher both pre- and postoperative complement activation and elevated postoperative cytokines (IL-6, IL-1ra, and MIP-1β) in PTA recipients with versus without early graft thrombosis. This pro-inflammatory pattern may indicate an amplified thromboinflammation in patients experiencing graft thrombosis. However, we cannot conclude if this pattern is causative or caused by venous thrombosis, but it supports the notion that inflammation plays a central role in venous thrombosis (44).

Current immunosuppressive therapies used in transplantation are mainly designed to affect the adaptive and not the innate immune response and thus lead to reduced production of pro- and anti-inflammatory cytokines, but do not inhibit the secretion of readily produced cytokines nor complement activation (45, 46). Complement inhibition has been suggested as a therapeutic option for ischemia-reperfusion injury in transplantation (47). Treatment with eculizumab, a complement blocker, that blocks cleavage of C5 and thus prevents C5a and C5b-9 generation, has reduced thrombosis rates in patients with paroxysmal nocturnal hematuria (48) and atypical hemolytic uremic syndrome (49). These conditions are chronic and require life-long treatment, while the transplantation setting represents a more temporary imbalance in complement system regulation. Presumably, a short complement inhibition may therefore reduce ischemia/reperfusion injury induced inflammation and risk of thrombosis.

This study has several limitations. Relatively few patients were included, especially for the subgroup analyses, which affects the power. On the other hand, this study included a high proportion of PTA recipients, and allowed for a comparison in the thromboinflammatory response between SPK and PTA recipients, which to our knowledge has not previously been performed. This is a single center-study with well-known limitations as to the generalizability of the results, but with the advantage that similar conditions for surgical technique, treatment, and follow-up were applied to all patients. We acknowledge that the graft thrombosis rate in this study is high. This most likely reflects the postoperative imaging protocol leading to an increased detection of partial graft thrombi that otherwise would have gone unnoticed. However, it enabled us to correlate inflammatory markers to any thrombotic event, rather than limiting the study to only include occluding thrombi.

We have some potential confounding factors. Anticoagulation was intensified during the study period, which may have affected thromboinflammation (50–52). However, to what extent is uncertain, and we demonstrated similar trends in inflammatory response between PTA and SPK recipients both before and after the anticoagulation change. Relaparotomy could increase thromboinflammation. However, no patients with early graft thrombosis underwent relaparotomy. The PTA and SPK populations differed pre- and perioperatively, which might have affected the postoperative outcome. PTA recipients had higher BMI and lower pancreas artery flow, factors that are putative risk factors for pancreas graft thrombosis (10). Indeed, BMI was identified as a risk factor for graft thrombosis. However, TCC concentrations on the first postoperative day remained a contributing risk factor when adjusted for BMI highlighting the importance of thromboinflammation. In addition, the higher hemoglobin concentration and lower pancreas artery flow observed in PTA recipients could increase the risk of thrombosis (53) but did not seem to be a contributing factor in our cohort when adjusted for the effect of transplantation recipient type (SPK/PTA). The study is descriptive and the results and their relevance for clinical outcomes need to be confirmed by further studies.

## Conclusion

Postoperatively, we found a relatively higher increase in the thromboinflammatory response in PTA recipients and higher absolute cytokine concentrations in SPK recipients.

CRP-increase and complement activation were associated with an increased risk of graft thrombosis in the early postoperative phase. TCC was more specific for graft thrombosis compared to CRP. Based on these findings, we suggest that the thromboinflammatory response in general, and complement activation, in particular, could be considered as contributing or as synergetic risk factors for early pancreas graft thrombosis.

## Data availability statement

The full datasets generated and analyzed in the current study are not publicly available due to Norwegian National Legislation prohibiting the publishing of information that could compromise research participant privacy. The collected data are of such nature that it falls under the provision of the Norwegian Health Research Act. However, an anonymized dataset of the inflammatory markers are available upon request to the corresponding authors. Additional data are available from Oslo University hospital’s data protection officer for research upon reasonable request. Researchers who meet the criteria for access to confidential data should make contact *via* personvern@ous-hf.no to receive access. Requests to access the datasets should be directed to Tor Åsmund Martinsen, personvern@ous-hf.no.

## Ethics statement

The studies involving human participants were reviewed and approved by South-Eastern Norwegian ethical committee. The patients/participants provided their written informed consent to participate in this study.

## Author contributions

All authors contributed substantially and followed the guidelines of the International Committee of Medical Journal Editors. KR, TM, HH, and SP participated in the study conception and research design, statistical analysis and interpretation and writing of the article. KR, GK, and HH collected the data. JK and TM performed the analyses. GK, RH, JK, TJ, P-DL, and TT reviewed and edited the manuscript. KR, SP, and HH participated in visualization. SP, HH, TT, and TM participated in supervision. HH, SP, TT, and TM provided resources. All authors were responsible for final approval and accountability for all aspects of the work. All authors contributed to the article and approved the submitted version.

## Glossary

**Table:**

BMI	body mass index
CAU	complement arbitrary unit
CRP	C-reactive protein
EDTA	ethylenediaminetetraacetic acid
ELISA	enzyme-linked immunosorbent assay
G-CSF	granulocyte colony-stimulating factor
IL	interleukin
IP-10	interferon gamma-induced protein 10
IV	intravenously
LMWH	low molecular weight heparin
MCP-1	monocyte chemoattractant protein 1
MIP	macrophage inflammatory protein
pg	picogram
PO	per orally
POD	postoperative day
PTA	pancreas transplant alone
SC	subcutaneously
SPK	simultaneous pancreas-kidney
TAT	thrombin-antithrombin complex
TCC	terminal complement complex
TNF	tumor necrosis factor
