# Supplementary material for: Thromboinflammatory response is increased in pancreas transplant alone versus simultaneous pancreas-kidney transplantation and early pancreas graft thrombosis is associated with complement activation
Source: Front Immunol. 2023 Mar 29;14:1044444. doi: 10.3389/fimmu.2023.1044444 (PMC10090504; doi:10.3389/fimmu.2023.1044444)
Supplement: Supplementary file 2 [file Table_1.docx]

**Table S1. Differences in inflammatory markers between specific time-points during the first postoperative week in PTA and SPK recipients**

| **Parameter** | **1^st^ postop day- preoperative**  **Estimate^1^ [95% CI]**  ***p*-value** | | **1^st^ postop day- 7^th^ postop day**  **Estimate [95% CI]**  ***p*-value** | | **7^th^ postop day- preoperative**  **Estimate [95% CI]**  ***p*-value** | |
| --- | --- | --- | --- | --- | --- | --- |
|  | **PTA^2^** | **SPK** | **PTA** | **SPK** | **PTA** | **SPK** |
| **Acute phase protein** |  |  |  |  |  |  |
| CRP (mg/L) | 1.48 [1.34-1.63]  ***p*<0.001** | 1.28 [1.14-1.42]  ***p*<0.001** | -0.34 [-0.49- -0.20]  ***p*<0.001** | -0.38 [-0.53- -0.25]  ***p*<0.001** | 1.14 [0.99-1.29]  ***p*<0.001** | 0.89 [0.75-1.03]  ***p*<0.001** |
| **Coagulation (ug/L)** |  |  |  |  |  |  |
| TAT | 0.43 [0.28-0.58]  ***p*<0.001** | 0.30 [0.15-0.44]  ***p*<0.001** | -0.80 [-0.97- -0.64]  ***p*<0.001** | -0.47 [-0.62- -0.33]  ***p*<0.001** | -0.38 [-0.54- -0.21]  ***p*<0.001** | -0.18 [-0.33- 0.023]  ***p*=0.024** |
| **Complement (CAU/ml)** |  |  |  |  |  |  |
| C3bc | 0.22 [0.15-0.30]  ***p*<0.001** | 0.17 [0.098-0.24]  ***p*<0.001** | -0.21 [-0.30- -0.14]  ***p*<0.001** | -0.17 [-0.24- -0.093] ***p*<0.001** | 0.007 [-0.074-0.088] *p*=0.86 | 0.012 [-0.074-0.076] *p*<0.9 |
| TCC | 0.25 [0.17-0.34]  ***p*<0.001** | 0.17 [0.086-0.25] ***p*<0.001** | 0.009 [-0.08-0.098]  *p*=0.84 | 0.011 [-0.079-0.081]  *p*>0.9 | 0.26 [0.17-0.35]  ***p*<0.001** | 0.17 [0.083-0.25]  ***p*<0.001** |
| **Cytokines (pg/ml)** |  |  |  |  |  |  |
| TNF | 0.088 [-0.061-0.24] *p*=0.25 | 0.18 [0.028-0.32] ***p*=0.02** | -0.21 [-0.37- -0.041] ***p*=0.014** | -0.35 [-0.50- -0.21]  ***p*<0.001** | -0.12 [-0.28- 0.05] *p*=0.17 | -0.18 [-0.33- -0.023] ***p*=0.025** |
| IL-6 | 0.90 [0.66-1.14]  ***p*<0.001** | 0.66 [0.42-0.90]  ***p*<0.001** | -0.55 [-0.82- -0.29]  ***p*<0.001** | -0.44 [-0.68- -0.20]  ***p*<0.001** | 0.35 [0.078-0.62]  ***p*=0.009** | 0.22 [-0.036-0.47]  *p*=0.13 |
| IL-8 | 0.61 [0.44-0.77]  ***p*<0.001** | 0.54 [0.38-0.71]  ***p*<0.001** | -0.49 [-0.67- -0.31]  ***p*<0.001** | -0.56 [-0.73- -0.37]  ***p*<0.001** | 0.12 [-0.067-0.30]  *p*=0.22 | -0.23 [-0.19-0.15]  *p*=0.80 |
| IL-1ra | 1. [0.87-1.2]   ***p*<0.001** | 1.28 [1.10-1.46]  ***p*<0.001** | -0.91 [-1.1- -0.77]  ***p*<0.001** | -1.15 [-1.33- -0.99]  ***p*<0.001** | 0.14 [-0.063-0.34]  *p*=0.18 | 0.13 [-0.063-0.32]  *p*=0.19 |
| IL-10 | 0.64 [0.33-0.95]  ***p*<0.001** | 0.45 [0.15-0.76]  ***p*=0.004** | -1.12 [-1.46- -0.79]  ***p*<0.001** | -0.82 [-1.13- -0.51]  ***p*<0.001** | -0.48 [-0.82- -0.14]  ***p*=0.005** | -0.36 [-0.68- -0.048]  ***p*=0.024** |
| IL-4 | 0.12 [0.032-0.20]  ***p*=0.007** | -0.076 [-0.16-0.0076]  *p*=0.075 | -0.022 [-0.11- 0.070]  *p*=0.64 | 0.080 [-0.0033-0.16]  *p*=0.06 | 0.094 [0.0006-0.19]  ***p*=0.049** | 0.0044 [-0.083-0.092]  *p*>0.9 |
| G-CSF | 0.79 [0.42-1.15]  ***p*<0.001** | 0.40 [0.037-0.77]  ***p*=0.031** | -0.57 [-0.97- -0.16]  ***p*=0.007** | -0.62 [-0.99- -0.21]  ***p*=0.001** | 0.22 [-0.19- 0.63]  *p*=0.29 | -0.22 [-0.60-0.16]  *p*=0.26 |
| IP-10 | 1.28 [1.14-1.42]  ***p*<0.001** | 1.0 [0.87-1.15]  ***p*<0.001** | -0.95 [-1.10- -0.79]  ***p*<0.001** | -0.81 [-0.95- -0.67]  ***p*<0.001** | 0.34 [0.18-0.50]  ***p*<0.001** | 0.20 [0.051-0.35]  ***p*=0.008** |
| MCP-1 | 0.85 [0.61-1.1]  ***p*<0.001** | 0.27 [0.035-0.51]  ***p*=0.024** | -0.64 [-0.91- -0.38]  ***p*<0.001** | -0.25 [-0.49- -0.016]  ***p*=0.036** | 0.20 [-0.06- 0.47]  *p*=0.49 | 0.017 [-0.23-0.26]  p=0.89 |
| MIP-1α | 0.34 [0.13-0.55]  ***p*=0.002** | 0.29 [0.082-0.50]  ***p*=0.006** | -0.30 [-0.53- -0.068]  ***p*=0.011** | -0.27 [-0.48- -0.062]  ***p*=0.011** | 0.037 [-0.19-0.27]  *p*=0.75 | 0.020 [-0.20-0.24]  *p*=0.65 |
| MIP-1β | -0.012 [-0.15-0.13] *p*=0.87 | -0.11 [-0.25-0.034]  *p*=0.14 | -0.28 [-0.44- -0.13]  ***p*<0.001** | -0.023 [-0.16-0.12]  *p*=0.75 | -0.30 [-0.45- -0.14]  ***p*<0.001** | -0.13 [-0.28-0.017]  *p*=0.083 |
| IL-5 | 0.17 [-0.0077-0.35]  *p*=0.06 | 0.032 [-0.14-0.21]  *p*=0.72 | -0.23 [-0.42- -0.035]  ***p*=0.021** | -0.095 [-0.27-0.082]  *p*=0.29 | -0.06 [-0.26-0.14]  *p*=0.55 | -0.062 [-0.24-0.12]  *p*=0.50 |
| IL-7 | 0.14 [-0.056-0.34]  *p*=0.16 | -0.058 [-0.25-0.14]  *p*=0.56 | 0.27 [0.048-0.48]  ***p*=0.017** | 0.35 [0.16-0.55]  ***p*<0.001** | 0.41 [0.19-0.63]  ***p*<0.001** | 0.30 [0.092-0.50]  ***p*=0.005** |
| IL-15 | 0.017 [-0.22-0.25]  *p*=0.89 | -0.42 [-0.65- -0.19]  ***p*<0.001** | 0.0020 [-0.25-0.26]  ***p*=0.006** | 0.22 [-0.013-0.45]  *p*=0.064 | 0.018 [-0.24-0.28]  *p*=0.89 | -0.20 [-0.44-0.41]  *p*=0.11 |

^1^ Estimates refer to mean differences estimated by the mixed model with log-transformed data. CI is the 95% confidence interval of the estimated group difference.

^2^ Abbreviations: CAU, complement arbitrary unit; CRP, C-reactive protein; G-CSF, granulocyte colony stimulating factor; IL, interleukin; IL-1ra: interleukin-1 receptor antagonist; IP-10, interferon gamma-induced protein 10; MCP-1, monocyte chemoattractant protein 1; MIP, macrophage inflammatory protein; PTA, Pancreas transplantation alone; SPK, Simultaneous pancreas-kidney transplantation; TAT, thrombin-antithrombin complex; TCC, terminal complement complex; TNF, tumour necrosis factor.
