# Supplementary material for: Thromboinflammatory response is increased in pancreas transplant alone versus simultaneous pancreas-kidney transplantation and early pancreas graft thrombosis is associated with complement activation
Source: Front Immunol. 2023 Mar 29;14:1044444. doi: 10.3389/fimmu.2023.1044444 (PMC10090504; doi:10.3389/fimmu.2023.1044444)
Supplement: Supplementary file 3 [file Table_2.docx]

**Table S2. Overall effects of group, time, and the interaction of group-by-time during the first postoperative week for the inflammatory parameters^1^**

| **Parameter** | **Group** | | **Time** | | **Interaction**  **(group-by-time)** | | |
| --- | --- | --- | --- | --- | --- | --- | --- |
|  | **Wald chi2** | ***p-*value** | **Wald chi2** | ***p-*value** | **Wald chi2** | ***p-*value** |  |
| **Acute phase protein** |  |  |  |  |  |  |  |
| CRP^2^ | 1.22 | 0.27 | 238.83 | **<0.001** | 250.68 | **<0.001** |  |
| **Coagulation** |  |  |  |  |  |  |  |
| TAT | 0.01 | >0.9 | 343.1 | **<0.001** | 382.52 | **<0.001** |  |
| **Complement** |  |  |  |  |  |  |  |
| C3bc | 0.68 | 0.41 | 97.2 | **<0.001** | 101.16 | **<0.001** |  |
| TCC | 0.01 | >0.9 | 3.23 | 0.78 | 5.86 | 0.95 |  |
| **Cytokines** |  |  |  |  |  |  |  |
| TNF | 4.66 | **0.031** | 166.55 | **<0.001** | 181.88 | **<0.001** |  |
| IL-6 | 2.88 | 0.090 | 105.17 | **<0.001** | 114.12 | **<0.001** |  |
| IL-8 | 17.4 | **<0.001** | 319.71 | **<0.001** | 342.96 | **<0.001** |  |
| IL-1ra | 11.53 | **<0.001** | 610.59 | **<0.001** | 649.99 | **<0.001** |  |
| IL-10 | 0.02 | 0.88 | 231.29 | **<0.001** | 260.66 | **<0.001** |  |
| IL-4 | 5.11 | **0.024** | 47.24 | **<0.001** | 65.75 | **<0.001** |  |
| G-CSF | 0.00 | >0.9 | 87.09 | **<0.001** | 95.9 | **<0.001** |  |
| IP-10 | 1.31 | 0.25 | 599.3 | **<0.001** | 626.11 | **<0.001** |  |
| MCP-1 | 1.43 | 0.23 | 56.88 | **<0.001** | 89.33 | **<0.001** |  |
| MIP-1α | 5.76 | **0.016** | 161.18 | **<0.001** | 175.91 | **<0.001** |  |
| MIP-1β | 0.86 | 0.35 | 89.45 | **<0.001** | 113.62 | **<0.001** |  |
| IL-5 | 3.25 | 0.072 | 47.57 | **<0.001** | 55.12 | **<0.001** |  |
| IL-7 | 0.59 | 0.44 | 34.07 | **<0.001** | 38.5 | **<0.001** |  |
| IL-15 | 0.08 | 0.77 | 9.87 | 0.13 | 20.82 | 0.077 |  |

^1^ Linear mixed model analyses on log-transformed data with either group (PTA/SPK), time, or group-by-time as independent variables. Overall effects determined with Wald Chi Squared test.

^2^ Abbreviations: CAU, complement arbitrary unit; CRP, C-reactive protein; G-CSF, granulocyte colony stimulating factor; IL, interleukin; IL-1ra: interleukin-1 receptor antagonist; IP-10, interferon gamma-induced protein 10; MCP-1, monocyte chemoattractant protein 1; MIP, macrophage inflammatory protein; PTA, Pancreas transplantation alone; SPK, Simultaneous pancreas-kidney transplantation; TAT, thrombin-antithrombin complex; TCC, terminal complement complex; TNF, tumour necrosis factor.
