# Supplementary material for: Thromboinflammatory response is increased in pancreas transplant alone versus simultaneous pancreas-kidney transplantation and early pancreas graft thrombosis is associated with complement activation
Source: Front Immunol. 2023 Mar 29;14:1044444. doi: 10.3389/fimmu.2023.1044444 (PMC10090504; doi:10.3389/fimmu.2023.1044444)
Supplement: Supplementary file 4 [file Table_3.docx]

**Table S3. SPK compared to PTA recipients at postoperative day 1, 2 and 7**

| **Parameter** | **1^st^ postoperative day**  **Estimate^1^ [95% CI]**  ***p*-value** | **2^nd^ postoperative day**  **Estimate [95% CI]**  ***p*-value** | **7^th^ postoperative day**  **Estimate [95% CI]**  ***p*-value** |
| --- | --- | --- | --- |
| **Acute phase protein** |  |  |  |
| CRP^2^ (mg/L) | 0.027 [-0.16-0.21]  *p*=0.77 | -0.018 [-0.21-0.17]  *p*=0.85 | -0.017 [-.0.21-0.17]  *p*=0.86 |
| **Coagulation (ug/L)** |  |  |  |
| TAT | -0.21 [-0.37- -0.56]  ***p*=0.008** | -0.0039 [-0.16- 0.15]  *p*>0.9 | 0.12 [-0.060-0.30]  *p*=0.18 |
| **Complement (CAU/ml)** |  |  |  |
| C3bc | -0.056 [-0.15-0.035]  *p*=0.23 | -0.049 [-0.14-0.042]  *p*=0.29 | -0.0089 [-0.11- 0.091]  *p*=0.86 |
| TCC | -0.00047 [-0.12-0.11]  *p*>0.9 | -0.023 [-0.14-0.092]  *p*=0.70 | -0.0081 [-0.13-0.12]  *p*=0.90 |
| **Cytokines (pg/ml)** |  |  |  |
| TNF | 0.24 [0.018-0.47]  ***p*=0.034** | 0.17 [-0.053-0.40]  *p*=0.13 | 0.095 [-0.15-0.34]  *p*=0.44 |
| IL-6 | 0.11 [-0.19-0.42]  *p*=0.47 | 0.072 [-0.23-0.37]  *p*=0.64 | 0.22 [-0.11-0.56]  *p*=0.19 |
| IL-8 | 0.23 [0.055-0.14]  ***p*=0.010** | 0.25 [0.071-0.43]  ***p*=0.006** | 0.16 [-0.043-0.36]  *p*=0.12 |
| IL-1ra | 0.41 [0.21-0.61]  ***p*<0.001** | 0.25 [0.045-0.45]  ***p*=0.016** | 0.17 [-0.063-0.39]  *p*=0.15 |
| IL-10 | -0.20 [-0.54-0.15]  *p*=0.27 | -0.20 [-0.54-0.15]  *p*=0.26 | 0.11 [-0.28-0.50]  *p*=0.57 |
| IL-4 | 0.033 [-0.093-0.16]  *p*=0.61 | 0.056 [-0.070-0.18]  *p*=0.38 | 0.14 [0.0000095-0.27]  *p*=0.050 |
| G-CSF | -0.033 [-0.50-0.43]  *p*=0.89 | -0.21 [-0.66-0.26]  *p*=0.39 | -0.089 [-0.60-0.42]  *p*=0.73 |
| IP-10 | -0.068 [-0.23-0.099]  *p*=0.43 | 0.13 [-0.041-0.29]  *p*=0.14 | 0.069 [-0.12-0.26]  *p*=0.46 |
| MCP-1 | -0.20 [-0.48-0.092]  *p*=0.18 | 0.12 [-0.17-0.40]  *p*=0.42 | 0.19 [-0.12-0.51]  *p*=0.23 |
| MIP-1α | 0.10 [-0.11-0.31]  *p*=0.36 | 0.55 [-0.16-0.27]  *p*=0.61 | 0.13 [-0.12-0.37]  *p*=0.30 |
| MIP-1β | -0.099 [-0.27-0.076]  *p*=0.27 | 0.054 [-0.12-0.23]  *p*=0.55 | 0.16 [-0.031-0.35]  *p*=0.10 |
| IL-5 | 0.014 [-0.20-0.23]  *p*=0.89 | 0.094 [-0.12-0.30]  *p*=0.38 | 0.15 [-0.085-0.38]  *p*=0.21 |
| IL-7 | 0.11 [-0.16-0.37]  *p*=0.43 | -0.029 [-0.29-0.23]  *p*=0.83 | 0.20 [-0.093-0.48]  *p*=0.18 |
| IL-15 | -0.25 [-0.48- -0.021]  ***p*=0.033** | 0.14 [-0.088-0.37]  *p*=0.23 | -0.034 [-0.30-0.23]  *p*=0.80 |

^1^ Estimates refer to mean group differences estimated by the mixed model with log-transformed data, CI is the 95% confidence interval of the estimated group difference,

^2^ Abbreviations: CAU, complement arbitrary unit; CRP, C-reactive protein; G-CSF, granulocyte colony stimulating factor; IL, interleukin; IL-1ra: interleukin-1 receptor antagonist; IP-10, interferon gamma-induced protein 10; MCP-1, monocyte chemoattractant protein 1; MIP, macrophage inflammatory protein; PTA, Pancreas transplantation alone; SPK, Simultaneous pancreas-kidney transplantation; TAT, thrombin-antithrombin complex; TCC, terminal complement complex; TNF, tumour necrosis factor
