# Supplementary material for: Thromboinflammatory response is increased in pancreas transplant alone versus simultaneous pancreas-kidney transplantation and early pancreas graft thrombosis is associated with complement activation
Source: Front Immunol. 2023 Mar 29;14:1044444. doi: 10.3389/fimmu.2023.1044444 (PMC10090504; doi:10.3389/fimmu.2023.1044444)
Supplement: Supplementary file 5 [file Table_4.docx]

**Table S4. Overall effects of group (PTA/SPK) in each anticoagulation group^1^**

| **Parameter** | **Anticoagulation group 1** | | **Anticoagulation group 2** | | |
| --- | --- | --- | --- | --- | --- |
|  | **Wald chi2** | ***p-*value** | **Wald chi2** | ***p-*value** |  |
| **Acute phase protein** |  |  |  |  |  |
| CRP^2^ | 0.09 | 0.76 | 0.36 | 0.55 |  |
| **Coagulation** |  |  |  |  |  |
| TAT | 2.13 | 0.14 | 0.52 | 0.47 |  |
| **Complement** |  |  |  |  |  |
| C3bc | 0.00 | >0.9 | 1.29 | 0.26 |  |
| TCC | 0.35 | 0.56 | 0.00 | >0.9 |  |
| **Cytokines** |  |  |  |  |  |
| TNF | 2.74 | 0.10 | 2.05 | 0.15 |  |
| IL-6 | 2.79 | 0.09 | 2.76 | 0.097 |  |
| IL-8 | 5.68 | **0.017** | 14.10 | **0.0002** |  |
| IL-1ra | 5.54 | **0.019** | 7.13 | **0.0076** |  |
| IL-10 | 2.60 | 0.11 | 1.19 | 0.28 |  |
| IL-4 | 10.7 | **0.0011** | 1.86 | 0.17 |  |
| G-CSF | 2.36 | 0.12 | 1.89 | 0.17 |  |
| IP-10 | 2.33 | 0.13 | 1.75 | 0.18 |  |
| MCP-1 | 0.00 | >0.9 | 3.01 | 0.083 |  |
| MIP-1α | 1.07 | 0.30 | 4.87 | **0.027** |  |
| MIP-1β | 0.39 | 0.54 | 0.10 | 0.75 |  |
| IL-5 | 2.24 | 0.13 | 2.20 | 0.13 |  |
| IL-7 | 4.78 | **0.029** | 0.06 | 0.80 |  |
| IL-15 | 0.11 | 0.74 | 1.53 | 0.22 |  |

^1^ Linear mixed model analyses on log-transformed data with anticoagulation group (first 34 patients versus the following 33 patients) as independent variable. Overall effects determined with Wald Chi Squared test. Significantly higher values in the SPK group for IL-8, IL-1ra, IL-4 and IL-7 in anticoagulation group1 and for IL-1ra, IL-8 and MIP-1α in the anticoagulation group 2.

^2^ Abbreviations: CAU, complement arbitrary unit; CRP, C-reactive protein; G-CSF, granulocyte colony stimulating factor; IL, interleukin; IL-1ra: interleukin-1 receptor antagonist; IP-10, interferon gamma-induced protein 10; MCP-1, monocyte chemoattractant protein 1; MIP, macrophage inflammatory protein; PTA, Pancreas transplantation alone; SPK, Simultaneous pancreas-kidney transplantation; TAT, thrombin-antithrombin complex; TCC, terminal complement complex; TNF, tumour necrosis factor.
