# Supplementary material for: Thromboinflammatory response is increased in pancreas transplant alone versus simultaneous pancreas-kidney transplantation and early pancreas graft thrombosis is associated with complement activation
Source: Front Immunol. 2023 Mar 29;14:1044444. doi: 10.3389/fimmu.2023.1044444 (PMC10090504; doi:10.3389/fimmu.2023.1044444)
Supplement: Supplementary file 6 [file Table_5.docx]

**Table S5. Fold changes from preoperative values to the first postoperative day, and sum of fold changes over the first postoperative week in PTA and SPK recipients^1^**

|  | **PTA** | | **SPK** | | **Comparison PTA vs SPK**  **fold change POD 1** | | **PTA** | **SPK** | **Comparison PTA vs SPK**  **sum of fold changes POD 1-7** |
| --- | --- | --- | --- | --- | --- | --- | --- | --- | --- |
| **Marker** | **Fold change**  **POD 1** | ***p-*value^2^** | **Fold**  **Change**  **POD 1** | ***p*-value^2^** | | ***p*-value^3^** | **Fold change sum**  **POD 1-7** | **Fold change sum**  **POD 1-7** | ***p*-value^3^** |
| CRP^4^ | 38.3  [10.1-79.8] | **<0.001** | 24.7  [8.71-63.8] | **<0.001** | 0.34 | | 221  [114-351] | 130  44-239] | 0.05 |
| TAT | 3.34  [1.60-6.72] | **0.004** | 2.43  [1.08-5.45] | **0.009** | 0.34 | | 8.22  [0.96-15.14] | 5.42  [0.51-14.73] | 0.32 |
| C3bc | 1.74  [0.97-2.53] | **0.036** | 1.41  [1.0-2.42] | **0.029** | 0.57 | | 8.96  [4.74-11.08] | 5.49  [1.87-9.58] | 0.15 |
| TCC | 1.51  [1.0-2.23] | **<0.001** | 1.50  [1.0-1.98] | **<0.001** | 0.72 | | 10.4  [7.20-14.78] | 7.18  [3.49-10.25] | **0.031** |
| TNF | 2.24  [0.55-4.27] | 0.052 | 2.42  [0.80-4.21] | **0.043** | 0.93 | | 9.07  [1.37-15.97] | 4.86  [0.26-11.35] | 0.18 |
| IL-6 | 15.44  [3.88-30.13] | **<0.001** | 5.48  [2.05-10.93] | **<0.001** | **0.013** | | 47.8  [9.06-106.5] | 10.3  [1.47-30.92] | **0.0092** |
| IL-8 | 6.90  [2.90-11.63] | **<0.001** | 5.18  [4.14-8.32] | **<0.001** | 0.34 | | 21.2  [7.80-38.52] | 13.16  [0.31-20.1] | **0.028** |
| IL-1ra | 8.75  [3.21-59.32] | **<0.001** | 31.24  [12.57-44.35] | **<0.001** | 0.16 | | 19.9  [4.22-76.54] | 28.4  [4.25-52.4] | 0.84 |
| IL-10 | 6.8  [2.20-51.25] | **0.002** | 6.19  [1.14-13.60] | **0.0015** | 0.31 | | 12.9  [1.0-54.8] | 5.84  [0.042-30.78] | 0.20 |
| IL-4 | 1.36  [0.94-1.90] | 0.11 | 0.83  [0.63-1.07] | 0.31 | **0.004** | | 7.49  [3.75-12.3] | 3.56  [1.25-8.42] | **0.024** |
| G-CSF | 15.36  [2.24-58.64] | **0.013** | 3.67  [0.67-17.38] | 0.17 | 0.12 | | 46.3  [0.78-92.6] | 7.95  [0.10-29.2] | **0.042** |
| IP-10 | 22.17  [11.63-43.55] | **<0.001** | 9.99  [7.21-21.9] | **<0.001** | **0.03** | | 45.0  [13.7-72.1] | 18.1  [7.30-36.49] | **0.0092** |
| MCP-1 | 6.64  [3.32-36.09] | **<0.001** | 2.93  [1.42-4.29] | **<0.001** | **0.015** | | 30.0  [6.30-59.8] | 11.8  [1.22-21.0] | **0.015** |
| MIP-1α | 5.35  [1.56-16.41] | **0.004** | 2.92  [1.58-4.80] | **<0.001** | 0.099 | | 19.3  [0.54-52.95] | 8.0  [0.096-18.0] | **0.044** |
| MIP-1β | 1.45  [0.70-2.70] | 0.09 | 1.08  [0.67-1.17] | 0.85 | 0.11 | | 4.95  [0.99-9.73] | 4.09  [0.39-7.30] | 0.19 |
| IL-5 | 2.13  [0.73-3.72] | 0.17 | 1.32  [0.56-2.31] | 0.54 | 0.34 | | 6.05  [2.45-17.05] | 4.04  [1.38-11.69] | 0.13 |
| IL-7 | 1.38  [0.74-3.07] | 0.31 | 0.75  [0.40-1.52] | 0.15 | 0.085 | | 17.6  [7.33-26.9] | 6.55  [2.97-14.1] | **0.016** |
| IL-15 | 0.82  [0.44-1.83] | 0.7 | 0.33  [0.13-0.77] | **0.009** | **0.022** | | 4.79  [2.31-9.73] | 3.24  [0.65-6.10] | 0.15 |

^1^ Median [25^th^-75^th^ percentile] fold change POD 1 and sum of fold changes POD 1-7 presented per inflammatory parameter and group (PTA/SPK).

^2^ Sign’s test used to test if fold changes within each group were significant.

^3^ Mann Whitney U-test for comparison between groups.

^4^ Abbreviations: CAU, complement arbitrary unit; CRP, C-reactive protein; G-CSF, granulocyte colony stimulating factor; IL, interleukin; IL-1ra: interleukin-1 receptor antagonist; IP-10, interferon gamma-induced protein 10; MCP-1, monocyte chemoattractant protein 1; MIP, macrophage inflammatory protein; PTA, Pancreas transplantation alone; SPK, Simultaneous pancreas-kidney transplantation; TAT, thrombin-antithrombin complex; TCC, terminal complement complex; TNF, tumour necrosis factor
