# Supplementary material for: Thromboinflammatory response is increased in pancreas transplant alone versus simultaneous pancreas-kidney transplantation and early pancreas graft thrombosis is associated with complement activation
Source: Front Immunol. 2023 Mar 29;14:1044444. doi: 10.3389/fimmu.2023.1044444 (PMC10090504; doi:10.3389/fimmu.2023.1044444)
Supplement: Supplementary file 7 [file Table_6.docx]

**Table S6. Logistic regression analyses of a thrombosis diagnosis, effect of inflammatory markers preoperatively and on the first postoperative day (all patients) ^1^**

| **Parameter** | **Preoperative values** | | **Values on the first postoperative day** | |
| --- | --- | --- | --- | --- |
|  | **OR [95% CI]** | ***p-*value** | **OR [95% CI]** | ***p-*value** |
| **Acute phase protein** |  |  |  |  |
| CRP^2^ (mg/L) (increase with 10) | 1.03 [0.99-1.07] | 0.16 | 1.16 [95% CI 1.01-1.34]^3^ | **0.042** |
| **Coagulation (ug/L)** |  |  |  |  |
| TAT | 1.00 [0.98-1.00] | 0.71 | 1.00 [0.99-1.00] | 0.62 |
| **Complement (CAU/ml)** |  |  |  |  |
| C3bc | 1.08 [0.87-1.34] | 0.51 | 1.06 [0.95-1.17] | 0.29 |
| TCC (increase with 0.1) | 14.8 [0.48-460] | 0.12 | 1.23 [95% CI 1.04-1.47] ^4^ | **0.019** |
| **Cytokines (pg/ml)** |  |  |  |  |
| TNF | 1.00 [1.00-1.00] | 0.60 | 1.00 [0.99-1.00] | 0.57 |
| IL-6 | 1.00 [1.00-1.00] | 0.70 | 1.00 [1.00-1.00] | 0.31 |
| IL-8 | 1.00 [1.00-1.00] | 0.84 | 0.99 [0.97-1.01] | 0.36 |
| IL-1ra | 1.00 [1.00-1.00] | 0.77 | 1.00 [1.00-1.00] | 0.24 |
| IL-10 | 1.00 [1.00-1.00] | 0.65 | 1.00 [1.00-1.00] | 0.37 |
| IL-4 | 0.96 [0.78-1.19] | 0.70 | 1.09 [0.94-1.55] | 0.26 |
| G-CSF | 1.00 [1.00-1.00] | 0.82 | 1.00 [1.00-1.00] | 0.42 |
| IP-10 | 1.00 [1.00-1.00] | 0.68 | 1.00 [1.00-1.00] | 0.65 |
| MCP-1 | 1.00 [1.00-1.00] | 0.49 | 1.00 [1.00-1.00] | 0.26 |
| MIP-1α | 1.00 [1.00-1.00] | 0.20 | 1.00 [0.92-1.05] | 0.67 |
| MIP-1β | 1.00 [1.00-1.00] | 0.25 | 1.00 [1.00-1.00] | 0.67 |
| IL-5 | 1.00 [0.98-1.01] | 0.58 | 1.02 [0.99-1.06] | 0.23 |
| IL-7 | 0.94 [0.88-1.04] | 0.25 | 1.01 [0.95-1.07] | 0.71 |
| IL-15 | 0.99 [0.96-1.01] | 0.22 | 1.01 [0.99-1.03] | 0.26 |

^1^Odds ratios (OR) presented with 95% confidence intervals (CI) and *p*-values for an increase in the inflammatory parameter of 1 unit if nothing else is indicated. The dependent variable is the diagnosis of a thrombosis within the first 30 postoperative days. Independent variables were concentrations of the different inflammatory variables preoperatively and on the first postoperative day.

^2^ Abbreviations: CAU, complement arbitrary unit; CRP, C-reactive protein; G-CSF, granulocyte colony stimulating factor; IL, interleukin; IL-1ra: interleukin-1 receptor antagonist; IP-10, interferon gamma-induced protein 10; MCP-1, monocyte chemoattractant protein 1; MIP, macrophage inflammatory protein; PTA, Pancreas transplantation alone; SPK, Simultaneous pancreas-kidney transplantation; TAT, thrombin-antithrombin complex; TCC, terminal complement complex; TNF, tumour necrosis factor.

^3^ An increase in CRP with 1 gives an OR of 1.01 [1.00-1.03] ^3^on the first postoperative day.

^4^ An increase in TCC with 1 CAU/ml gives an OR of 8.0 [1.4-46] on the first postoperative day.
