# Supplementary material for: Thromboinflammatory response is increased in pancreas transplant alone versus simultaneous pancreas-kidney transplantation and early pancreas graft thrombosis is associated with complement activation
Source: Front Immunol. 2023 Mar 29;14:1044444. doi: 10.3389/fimmu.2023.1044444 (PMC10090504; doi:10.3389/fimmu.2023.1044444)
Supplement: Supplementary file 8 [file Table_7.docx]

**Table S7. Univariate and multivariable logistic regression results for factors associated with pancreas graft thrombosis**

| **Factors** | **Univariate relative risk**  **OR [95% CI]** | ***P*-value** | **Multivariable relative risk adjusted for**  **group (PTA/SPK)**  **OR [95% CI]** | ***P*-value** |
| --- | --- | --- | --- | --- |
| Body mass index  (kg/m^2^) | 1.23[1.15-1.31] | <0.001 | 1.14[1.06-1.22] | <0.001 |
| Hemoglobin (g/dL) | 1.36[1.20-1.53] | <0.001 | 1.03[0.89-1.20] | 0.67 |
| Pancreas artery flow (ml/min) | 1.00[1.00-1.00] | 0.007 | 1.00[1.00-1.00] | 0.28 |

Odds ratios (OR) presented with 95% confidence intervals (CI) and *p*-values, for an increase in the factors with 1 unit.
