# Supplementary material for: Thromboinflammatory response is increased in pancreas transplant alone versus simultaneous pancreas-kidney transplantation and early pancreas graft thrombosis is associated with complement activation
Source: Front Immunol. 2023 Mar 29;14:1044444. doi: 10.3389/fimmu.2023.1044444 (PMC10090504; doi:10.3389/fimmu.2023.1044444)
Supplement: Supplementary file 9 [file Table_8.docx]

**Table S8. Multivariable logistic regression results for TCC on postoperative day 1 (increase of TCC with 0.1 CAU) adjusted for BMI, and group (PTA/SPK)**

| **Factors** | **Multivariable relative risk**  **OR [95% CI]** | ***P*-value** |
| --- | --- | --- |
| Body mass index | 1.5 [1.0 - 2.2]^1^ | 0.029 |
| Type of transplantation (PTA/SPK) | 1.3 [1.1 - 1.5] ^2^ | 0.009 |

^1^ An increase in TCC with 1 CAU/ml on the first postoperative day adjusted for BMI gives an OR of 7.77[1.23-49] and ^2^adjusted for the type of transplantation (PTA/SPK) an OR of 11.4[1.8-70.4].
TCC, terminal complement complex; CAU, complement arbitrary units; BMI, body mass index; PTA, pancreas transplant alone; SPK, simultaneous pancreas kidney; OR, odds ratio; CI, confidence interval
