# Supplementary material for: Thromboinflammatory response is increased in pancreas transplant alone versus simultaneous pancreas-kidney transplantation and early pancreas graft thrombosis is associated with complement activation
Source: Front Immunol. 2023 Mar 29;14:1044444. doi: 10.3389/fimmu.2023.1044444 (PMC10090504; doi:10.3389/fimmu.2023.1044444)
Supplement: Supplementary file 10 [file Table_9.docx]

**Table S9. Preoperative inflammatory parameter levels in patients with and without a pancreas graft thrombosis in the PTA group**

| **Parameter** | **Thrombosis**  **(N=14)** | **No thrombosis**  **(N=18)** | ***p*-value^1^** |
| --- | --- | --- | --- |
| **Acute phase protein** |  |  |  |
| CRP^2^ (mg/L) | 1.9 [0.9-9.1] | 0.7 [0.6-3.1] | 0.28 |
| **Coagulation (ug/L)** |  |  |  |
| TAT | 9.0 [4.9-31] | 10 [6.2-17] | 0.85 |
| **Complement (CAU/ml)** |  |  |  |
| C3bc | 4.0 [3.0-6.5] | 3.3 [2.8-5.5] | 0.57 |
| TCC | 0.30 [0.20-0.47] | 0.20 [0.10-0.30] | **0.038** |
| **Cytokines (pg/ml)** |  |  |  |
| TNF | 22 [15-32] | 20 [13-28] | 0.50 |
| IL-6 | 1.5 [0.77-5.7] | 0.70 [0.29-2.3] | 0.15 |
| IL-8 | 3.0 [1.0-4.9] | 1.8 [1.4-3.1] | 0.61 |
| IL-1ra | 223 [82-304] | 89 [24-146] | 0.067 |
| IL-10 | 2.0 [0.74-47] | 2.3 [1.0-6.7] | 0.89 |
| IL-4 | 1.6 [0.99-2.2] | 2.2 [1.5-2.6] | 0.34 |
| G-CSF | 7.9 [0.98-504] | 4.0 [1.2-42] | 0.72 |
| IP-10 | 428 [318-851] | 283 [191-390] | 0.062 |
| MCP-1 | 13.7 [8.6-21] | 9.9 [5.4-15] | 0.58 |
| MIP-1α | 2.2 [0.78-51] | 1.0 [0.49-3.6] | 0.15 |
| MIP-1β | 102 [86-782] | 88 [41-101] | 0.13 |
| IL-5 | 6.9 [3.4-15] | 3.4 [2.8-15] | 0.63 |
| IL-7 | 2.2 [6.0-85] | 3.1 [1.7-5.2] | >0.9 |
| IL-15 | 8.2 [6.0-20] | 22 [12-36] | 0.074 |

^1^ Median and quartiles are presented. Group comparisons were performed with Mann-Whitney U-test.

^2^ Abbreviations: CAU, complement arbitrary unit; G-CSF, granulocyte colony stimulating factor; IL, interleukin; IL-1ra: interleukin-1 receptor antagonist; IP-10, interferon gamma-induced protein 10; MCP-1, monocyte chemoattractant protein 1; MIP, macrophage inflammatory protein; PTA, Pancreas transplantation alone; TAT, thrombin-antithrombin complex; TCC, terminal complement complex; TNF, tumour necrosis factor.
