# Supplementary material for: Thromboinflammatory response is increased in pancreas transplant alone versus simultaneous pancreas-kidney transplantation and early pancreas graft thrombosis is associated with complement activation
Source: Front Immunol. 2023 Mar 29;14:1044444. doi: 10.3389/fimmu.2023.1044444 (PMC10090504; doi:10.3389/fimmu.2023.1044444)
Supplement: Supplementary file 11 [file Table_10.docx]

**Table S10. PTA recipients with and without pancreas graft thrombosis compared at postoperative day 1, 2 and 7**

| **Parameter** | **1^st^ postoperative day**  **Estimate^1^ [95% CI]**  ***p*-value** | **2^nd^ postoperative day**  **Estimate [95% CI]**  ***p*-value** | **7^th^ postoperative day**  **Estimate [95% CI]**  ***p*-value** |
| --- | --- | --- | --- |
| **Acute phase protein** |  |  |  |
| CRP^2^ (mg/ml) | 0.30 [0.028-0.57]  ***p*=0.031** | 0.21 [-0.060-0.48]  *p*=0.13 | -0.075 [-0.35-0.20]  *p*=0.59 |
| **Coagulation (ug/L)** |  |  |  |
| TAT | -0.15 [-0.37-0.080]  *p*=0.21 | -0.10 [-0.33-0.13]  *p*=0.39 | -0.11 [-0.39-0.16]  *p*=0.42 |
| **Complement (CAU/ml)** |  |  |  |
| C3bc | 0.039 [-0.10-0.18]  *p*=0.59 | -0.060 [-0.20-0.80]  *p*=0.40 | 0.011 [-0.15-0.17]  *p*=0.89 |
| TCC | 0.34 [0.18-0.50]  ***p*<0.001** | 0.14 [-0.023-0.29]  *p*=0.094 | 0.0034 [-0.18-0.19]  *p*>0.9 |
| **Cytokines (pg/ml)** |  |  |  |
| TNF | -0.088 [-0.41-0.23]  *p*=0.59 | -0.00084 [-0.32-0.32]  *p*>0.9 | 0.14 [-0.23-0.50]  *p*=0.46 |
| IL-6 | 0.64 [0.15-1.1]  ***p*=0.011** | 0.30 [-0.19-0.79]  *p*=0.24 | 0.29 [-0.28-0.86]  *p*=0.32 |
| IL-8 | 0.069 [-0.20-0.34]  *p*=0.61 | 0.10 [-0.17-0.37]  *p*=0.46 | 0.0048 [-0.33-0.34]  *p*>0.9 |
| IL-1ra | -0.25 [-0.56-0.061]  *p*=0.12 | 0.12 [-0.19-0.43]  *p*=0.45 | 0.26 [-0.12-0.63]  *p*=0.18 |
| IL-10 | -0.23 [-0.74-0.28]  *p*=0.37 | 0.16 [-0.34-0.67]  *p*=0.53 | 0.029[-0.58-0.64]  *p*>0.9 |
| IL-4 | 0.13 [-0.06-0.32]  *p*=0.18 | -0.033 [-0.22-0.16]  *p*=0.73 | 0.0079 [-0.21-0.22]  *p*>0.9 |
| G-CSF | -0.13 [-0.80-0.54]  *p*=0.71 | 0.053 [-0.62-0.72]  *p*=0.88 | 0.26 [-0.53-1.1]  *p*=0.52 |
| IP-10 | -0.15 [-0.41-0.10]  *p*=0.25 | 0.042 [-0.21-0.30]  *p*=0.75 | 0.20 [-0.11-0.51]  *p*=0.21 |
| MCP-1 | 0.22 [-0.22-0.66]  *p*=0.33 | 0.054 [-0.38-0.50]  *p*=0.81 | 0.22 [-0.31-0.75]  *p*=0.41 |
| MIP-1α | 0.062 [-0.28-0.41]  *p*=0.73 | 0.095 [-0.25-0.44]  *p*=0.59 | 0.21 [-0.22-0.64]  *p*=0.34 |
| MIP-1β | 0.11 [-0.17-0.39]  *p*=0.43 | 0.17 [-0.11-0.45]  *p*=0.22 | 0.21 [-0.12-0.54]  *p*=0.22 |
| IL-5 | 0.25 [-0.095-0.60]  *p*=0.15 | 0.28 [-0.067-0.63]  *p*=0.11 | 0.20 [-0.22-0.61]  *p*=0.35 |
| IL-7 | 0.13 [-0.25-0.51]  *p*=0.50 | -0.050 [-0.43-0.33]  *p*=0.80 | -0.80 [-1.3- -0.35]  ***p*<0.001** |
| IL-15 | -0.056 [-0.42-0.31]  *p*=0.76 | -0.10 [-0.47-0.26]  *p*=0.59 | -0.031 [-0.49-0.42]  *p*=0.89 |

^1^ Estimates refer to mean group differences estimated by the mixed model with log (10)-transformed data, CI is the 95% confidence interval of the estimated group difference,

^2^ Abbreviations: CAU, complement arbitrary unit; G-CSF, granulocyte colony stimulating factor; IL, interleukin; IL-1ra: interleukin-1 receptor antagonist; IP-10, interferon gamma-induced protein 10; MCP-1, monocyte chemoattractant protein 1; MIP, macrophage inflammatory protein; PTA, Pancreas transplantation alone; SPK, Simultaneous pancreas-kidney transplantation; TAT, thrombin-antithrombin complex; TCC, terminal complement complex; TNF, tumour necrosis factor.
