# Supplementary material for: Thromboinflammatory response is increased in pancreas transplant alone versus simultaneous pancreas-kidney transplantation and early pancreas graft thrombosis is associated with complement activation
Source: Front Immunol. 2023 Mar 29;14:1044444. doi: 10.3389/fimmu.2023.1044444 (PMC10090504; doi:10.3389/fimmu.2023.1044444)
Supplement: Supplementary file 12 [file Table_11.docx]

**Table S11. Overall effects of group (thrombosis/no thrombosis), time and the interaction of group-by-time during the first postoperative week in PTA recipients**

| **Parameter** | **Group** | | **Time** | | **Interaction^1^**  **(group-by-time)** | | |
| --- | --- | --- | --- | --- | --- | --- | --- |
|  | **Wald chi2** | ***p-*value** | **Wald chi2** | ***p-*value** | **Wald chi2** | ***p-*value** |  |
| **Acute phase protein** |  |  |  |  |  |  |  |
| CRP^2^ (mg/ml) | 0.86 | 0.35 | 94.73 | **<0.001** | 115.53 | **<0.001** |  |
| **Coagulation (ug/L)** |  |  |  |  |  |  |  |
| TAT | 3.05 | 0.081 | 301.86 | **<0.001** | 319.13 | **<0.001** |  |
| **Complement (CAU/ml)** |  |  |  |  |  |  |  |
| C3bc | 0.2 | 0.66 | 56.02 | **<0.001** | 62.03 | **<0.001** |  |
| TCC | 4.72 | **0.030** | 3.42 | 0.75 | 27.93 | **0.0092** |  |
| **Cytokines (pg/ml)** |  |  |  |  |  |  |  |
| TNF | 0.06 | 0.81 | 69.29 | **<0.001** | 76.2 | **<0.001** |  |
| IL-6 | 2.24 | 0.13 | 56.93 | **<0.001** | 69.56 | **<0.001** |  |
| IL-8 | 2.31 | 0.13 | 121.56 | **<0.001** | 125.94 | **<0.001** |  |
| IL-1ra | 3.73 | 0.053 | 212.28 | **<0.001** | 245.81 | **<0.001** |  |
| IL-10 | 0.01 | 0.92 | 159.53 | **<0.001** | 167.51 | **<0.001** |  |
| IL-4 | 0 | >0.9 | 22.61 | **0.0009** | 28.6 | **<0.001** |  |
| G-CSF | 0 | 0.96 | 43.49 | **<0.001** | 45.66 | **<0.001** |  |
| IP-10 | 0.78 | 0.38 | 308.27 | **<0.001** | 324.75 | **<0.001** |  |
| MCP-1 | 1.9 | 0.17 | 48.29 | **<0.001** | 51.06 | **<0.001** |  |
| MIP-1α | 5.84 | **0.016** | 77.31 | **<0.001** | 85.47 | **<0.001** |  |
| MIP-1β | 2.49 | 0.11 | 63.33 | **<0.001** | 70.11 | **<0.001** |  |
| IL-5 | 4.43 | **0.035** | 25.92 | **0.0002** | 31.74 | **0.0026** |  |
| IL-7 | 1.94 | 0.164 | 13.68 | **0.033** | 33.13 | **0.0016** |  |
| IL-15 | 0.65 | 0.42 | 10.99 | 0.089 | 12.35 | 0.50 |  |

^1^ Linear mixed model analyses on log-transformed data. Group or time or group-by-time as independent variables. Overall effects determined with Wald Chi Squared test.

^2^ Abbreviations: CAU, complement arbitrary unit; G-CSF, granulocyte colony stimulating factor; IL, interleukin; IL-1ra: interleukin-1 receptor antagonist; IP-10, interferon gamma-induced protein 10; MCP-1, monocyte chemoattractant protein 1; MIP, macrophage inflammatory protein; PTA, Pancreas transplantation alone; SPK, Simultaneous pancreas-kidney transplantation; TAT, thrombin-antithrombin complex; TCC, terminal complement complex; TNF, tumour necrosis factor.
