# Supplementary material for: Thromboinflammatory response is increased in pancreas transplant alone versus simultaneous pancreas-kidney transplantation and early pancreas graft thrombosis is associated with complement activation
Source: Front Immunol. 2023 Mar 29;14:1044444. doi: 10.3389/fimmu.2023.1044444 (PMC10090504; doi:10.3389/fimmu.2023.1044444)
Supplement: Supplementary file 13 [file Table_12.docx]

**Table S12. Univariate logistic regression analyses investigating the effect of inflammatory markers preoperatively and on the first postoperative day for pancreas transplant alone (PTA) recipients^1^ on risk for graft thrombosis**

| **Parameter** | **Preoperative values** | | **Values on the first postoperative day** | |
| --- | --- | --- | --- | --- |
|  | **OR [95% CI]** | ***p-*value** | **OR [95% CI]** | ***p-*value** |
| **Acute phase protein** |  |  |  |  |
| CRP^2^ (mg/ml) (increase with 10) | 1.20 [0.96-1.50] | 0.11 | 1.35 [95% CI 1.05-1.75] ^3^ | **0.021** |
| **Coagulation (ug/L)** |  |  |  |  |
| TAT | 1.00 [0.98-1.01] | 0.71 | 1.00 [0.99-1.00] | >0.9 |
| **Complement (CAU/ml)** |  |  |  |  |
| C3bc | 1.03 [0.77-1.36] | 0.86 | 1.06 [0.93-1.22] | 0.35 |
| TCC(increase with 0.1) | 221 [0.95-51600] | 0.052 | 1.4 [95% CI 1.0-2.0] ^4^ | **0.029** |
| **Cytokines (pg/ml)** |  |  |  |  |
| TNF | 1.00 [1.00-1.00] | 0.245 | 1.00 [0.99-1.01] | 0.83 |
| IL-6 | 1.00 [0.99-1.02] | 0.42 | 1.04 [0.99-1.09] | 0.10 |
| IL-8 | 1.00 [1.00-1.00] | 0.70 | 1.00 [0.98-1.03] | 0.31 |
| IL-1ra | 1.00 [1.00-1.00] | 0.93 | 1.00 [1.00-1.00] | 0.69 |
| IL-10 | 1.00 [1.00-1.00] | 0.47 | 1.00 [1.00-1.00] | 0.54 |
| IL-4 | 1.13 [0.74-1.76] | 0.57 | 1.12 [0.83-1.51] | 0.47 |
| G-CSF | 1.00 [1.00-1.00] | 0.44 | 1.00 [1.00-1.00] | 0.57 |
| IP-10 | 1.00 [1.00-1.00] | 0.44 | 1.00 [1.00-1.00] | 0.94 |
| MCP-1 | 1.00 [1.00-1.00] | 0.92 | 1.00 [1.00-1.00] | 0.41 |
| MIP-1α | 1.00 [1.00-1.00] | 0.48 | 1.03 [0.92-1.15] | 0.61 |
| MIP-1β | 1.00 [1.00-1.00] | 0.32 | 1.00 [1.00-1.00] | 0.41 |
| IL-5 | 1.00 [0.98-1.03] | >0.9 | 1.02 [0.98-1.07] | 0.34 |
| IL-7 | 1.10 [0.90-1.34] | 0.34 | 1.05 [0.94-1.18] | 0.87 |
| IL-15 | 1.00 [0.99-1.02] | 0.38 | 1.00 [0.99-1.02] | 0.67 |

^1^ Odds ratios (OR) presented with 95% confidence intervals (CI) and *p*-values. The dependent variable in this analysis is the diagnosis of a thrombus within the first 30 postoperative days. Independent variables were concentrations of the different inflammatory variables preoperatively and on the first postoperative day. The ORs are given for an increase in the concentration with 1 unit.

^2^ Abbreviations: CAU, complement arbitrary unit; G-CSF, granulocyte colony stimulating factor; IL, interleukin; IL-1ra: interleukin-1 receptor antagonist; IP-10, interferon gamma-induced protein 10; MCP-1, monocyte chemoattractant protein 1; MIP, macrophage inflammatory protein; PTA, Pancreas transplantation alone; SPK, Simultaneous pancreas-kidney transplantation; TAT, thrombin-antithrombin complex; TCC, terminal complement complex; TNF, tumour necrosis factor.

^3^ An increase in CRP with 1 mg/ml gives an OR of 1.03 [1.00-1.06] on the first postoperative day.

^4^ An increase in TCC with 1 CAU/ml gives an OR of 36.0 [1.43-900] on the first postoperative day.
